# β-Sitosterol β-d-glucoside (BSSG) triggers intestinal inflammation in zebrafish and mouse models prior to neurodegeneration onset

**DOI:** 10.1186/s12929-026-01249-8

**Published:** 2026-05-04

**Authors:** Francesca Terrin, Sofia Faggin, Edoardo Bizzotto, Davide Santinello, Silvia Cerantola, Giuseppe Borsato, Fabrizio Fabris, Alessandro Scarso, Rosario Licitra, Graziano Guella, Gabriele Sales, Stefano Cagnin, Laura Treu, Luigi Bubacco, Maria Cecilia Giron, Nicoletta Plotegher, Luisa Dalla Valle

**Affiliations:** 1 Department of Biology, University of Padova, Via U. Bassi 58/B, 35131 Padua, Italy; 2 Department of Pharmaceutical and Pharmacological Sciences, University of Padova, Largo Meneghetti 2, 35131 Padua, Italy; 3 Institute of Digestive Health Research (IRSD), INSERM U1022, INRAe, ENVT, Toulouse University, Toulouse, France; 4 Department of Molecular Science and Nanosystems, Università Ca’ Foscari Venezia, 30172 Mestre Venice, Italy; 5 Department of Veterinary Sciences, University of Pisa, 56124 Pisa, Italy; 6 Department of Biology, University of Trento, 38123 Trento, Italy; 7 CIR-Myo Myology Center, University of Padova, 35131 Padua, Italy

**Keywords:** BSSG, Glucosylated sterols, Intestinal inflammation, Gut microbiota, Glucocorticoid receptor, Gut–brain axis, Zebrafish model, Mouse model

## Abstract

**Background:**

Glucosylated-sterols can be synthetized endogenously, absorbed through the diet or derive from bacterial infection. Their clinical relevance is currently underestimated, even though their imbalance has been associated with an increased risk of neurodegeneration over the lifespan. We studied the detrimental effects elicited by dietary consumption of the plant-derived β-sitosterol β-d-glucoside (BSSG), known to be associated with the occurrence of ALS-PDC, to elucidate its potential mechanism of action.

**Methods:**

Zebrafish larvae and adults, as well as mice, were treated with BSSG administered directly in the water or via customized food pellet, respectively. Since the intestine was identified as the primary target tissue, its morphological and functional characteristics were assessed, together with transcriptional profiling and gut microbiota sequencing. Ex vivo analysis of zebrafish gut contractility was applied to evaluate intestinal neuromuscular responses. Mutant and transgenic zebrafish lines were used to explore a potential BSSG mechanism of action.

**Results:**

BSSG induced intestinal inflammation in both zebrafish and mouse models. This previously unknown effect was evidenced by gut dysmotility and inflammatory response. Transcriptomic analyses revealed increased expression of inflammation-related genes in the intestine of both zebrafish and mice, while preliminary gut microbiota analyses suggested the onset of dysbiosis. Transgenic and mutant zebrafish lines, depleted of genes involved in glucocorticoids synthesis and activity, evidenced that BSSG likely interacts with the glucocorticoid receptor, potentially impairing its canonical anti-inflammatory activity.

**Conclusions:**

We identified novel pathways altered by dietary BSSG exposure. This molecule appears to initially induce gut inflammation, leading to changes in intestinal morphology and function, and may contribute to neurodegeneration through disruption of the well-known gut–brain axis.

**Supplementary Information:**

The online version contains supplementary material available at 10.1186/s12929-026-01249-8.

## Background

Amyotrophic Lateral Sclerosis-Parkinsonism Dementia complex (ALS-PDC) is a rare neurodegenerative disorder characterized by symptoms resembling amyotrophic lateral sclerosis (ALS), including motor neurons loss and progressive muscle wasting, as well as Parkinsonian features and dementia in later stages [1]. ALS-PDC was first identified in the early 1960s among the indigenous population of Guam, and was later reported in the Kii peninsula of Honshu Island in Japan [2] and in Western New Guinea [3]. Intriguingly, populations living in these regions consumed flour derived from the seeds of cycad plants—*Cycas micronesica*, *C. revoluta*, and *C. circinalis*—as part of their diet and traditional medicine [4]. This shared dietary habit was therefore considered a primary environmental trigger of the disorder. Notably, after World War II, with the increasing adoption of Western habits and the reduced consumption of cycad-based products, the incidence of ALS-PDC declined significantly [5]. The key risk factor was identified as β-sitosterol β-D-glucoside (BSSG), a compound present in considerable concentration in cycad seeds. This molecule has been shown to be neurotoxic both in vitro and in vivo, inducing glutamate-mediated excitotoxicity, promoting hyperphosphorylated tau accumulation in neurons, and exacerbating apoptosis in cultured astrocytes [6, 7]. Therefore, elevated endogenous levels of BSSG, introduced through the diet, appeared to contribute to neurotoxicity and neuroinflammation, ultimately leading to the development of ALS-PDC [6].

BSSG is a glucosylated sterol composed of a steroid backbone linked to a glucose moiety. Similar molecules are predominantly found in plants, fungi and algae and rarely in bacteria and animals, where they occur as glucosylated forms of cholesterol [8]. Interestingly, increased levels of glucosyl-β-d-cholesterol (β-GlcChol), a glucosylated sterol endogenously synthetized in humans, have been found in patients harboring mutations in *GBA1* gene, a major genetic risk factors for Parkinson’s disease (PD) [9]. Similarly, the glucosyl-α-d-cholesterol (α-GlcChol) is produced by the bacterium *Helycobacter pylori* during gastric infections, a condition regarded as an environmental factor associated with increased lifetime risk of developing PD [10]. Despite their clinical relevance, the mechanisms by which glucosylated sterols dysregulation exerts deleterious effects on the nervous system remain poorly understood.

In this study, we employed zebrafish and mouse models to elucidate how BSSG may contribute to neurotoxicity. Unexpectedly, our findings indicate that the intestine represents the primary target tissue. We showed that this molecule induces sustained gut inflammation, characterized by altered intestinal physiology, motility and gene expression in both animal models. Given that BSSG-treated mice represent an established pre-symptomatic model for ALS-PDC [11] and in light of the intestinal inflammatory response observed here and previously unreported, we propose that dysregulation of the gut–brain axis may affect the physiological balance between the intestine and the brain.

Finally, we identified a potential interaction between BSSG and the glucocorticoid receptor (GR), likely mediated by its structural similarity to steroid molecules, thus suggesting a novel mechanism of action.

## Methods

### β-Sitosterol β-d-glucoside synthesis

β-Sitosterol β-d-glucoside (BSSG) synthesis process and chemical characterization are extensively described in Additional File 1.

### Zebrafish and mouse husbandry and treatment

Zebrafish wild type (WT) lines used in this work, including those used for the generation of the stable mutant lines, derived from Tuebingen and Giotto strains matings. Embryos, larvae and adults, housed in the Zebrafish Facility of the University of Padova, were maintained according to standard procedures [12]. Embryos were obtained from natural mating of WT, mutant, or transgenic adult fish and raised at 28.5 °C in Petri dishes with Fish Water (50×: 25 g Instant Ocean, 39.25 g CaSO_4_ and 5 g NaHCO_3_ in 1L) maintained in a 12 h light:12 h dark (LD) cycle until 72 h post fertilization (hpf). At this life stage, larvae were screened for fluorescence, when necessary, and exposed to treatment or control vehicle.

WT C57BL/6 male mice, housed in the Animal Facility of the University of Padova, were fed for 15 weeks, following weaning (1 month after birth) and up to 6 months of age, with BSSG-enriched chow pellet or commercial food as a control. Mice food pellet was prepared by Mucedola Srl. The treatment paradigm, previously established in [11], consisted in feeding mice daily with 1 mg/day of BSSG for 5 days/week. At the end of the experimentation period, mice were sacrificed by cervical dislocation, weighed and dissected for organs collection. All husbandry and experimental protocols were in accordance with national and EU guidelines for the use of experimental animals and were approved by the Animal Care and Use Ethics Committee of the University of Padua and by the Italian Ministry of Health (Authorization n. 112/2015PR; Authorization n. 12/2023-PR; Authorization n. 690/2020-PR).

### Chemicals preparation

BSSG powder (MW = 576.86 g/mol) was dissolved in DMSO (Sigma-Aldrich), while β-sitosterol powder (β-Sito, MW = 414.71 g/mol; 85451, Sigma-Aldrich) was dissolved in ethanol (EtOH), both at a final concentration of 10 mM, and sonicated until complete solubilization. Ultrasonic bath temperature was kept at around 26–27 °C. Stock aliquots were stored at − 20 °C. All chemicals were administered to zebrafish larvae diluted to a final working concentration of 10 µM, while control larvae were exposed to the same volume of the vehicle, reaching a maximum concentration of 0.1% v/v.

### Treatment of zebrafish larvae

For acute treatment, zebrafish larvae were incubated in Fish Water containing either BSSG, β-sitosterol or vehicle from 3 to 5 days post fertilization (dpf). Treatment was renewed every 24 h to avoid molecule deposits and changes in concentration.

Chronic 15-days-long BSSG treatment was performed on zebrafish larvae from 15 to 30 dpf. Briefly, larvae obtained from three independent spawns were raised in the Zebrafish Facility of the University of Padova according to standard procedures until 15 dpf. They were then divided into 3 treatment and 3 control groups and maintained during the experimentation period in glass beakers containing 300 mL of Fish Water at 28.5 °C under a 12:12 LD period and fed three times/day. Treatment was administered directly in the water to a final concentration of 10 µM and refreshed every 2 days.

### BSSG-enriched diet for adult zebrafish

The experimental BSSG-enriched feed was produced at the Department of Veterinary Sciences of the University of Pisa by incorporating the compound in a control feed, as described in [13]. Briefly, the control feed (a mixture of 50% TetraMin Flakes^®^ and 50% GEMMA Micro 300®) was first finely ground (< 100 µm) and then mixed with the BSSG powder to achieve a uniform dispersion. Distilled water was added to obtain a homogeneous dough, which was pelleted and dried at 40 °C in a drying chamber for 24 h. Finally, dry pellets were re-ground to restore feed particle size and kept at − 20 °C until use. Commercial feed without BSSG was processed using an identical procedure as a control. Male adult zebrafish at 4 months post fertilization (mpf), obtained from three independent spawns, were divided (*N* ≥ 4) into treatment and control groups, homogeneous in body weight. Fish were fed 5% of their body weight once per day, according to [14], to achieve a BSSG intake of approximately 15 µg/day/fish. The experiment lasted for 30 days.

### DNA extraction and genotyping of mutant zebrafish

Genotyping of mutant zebrafish was performed at early stages from genomic DNA extracted with the HotSHOT protocol [15] and amplified with locus-specific primers through PCR with 5× HOT FIREPol^®^ Blend Master Mix (04–25-00125, Solis BioDyne). Primers used for genotyping are listed in Additional file 2: Table 1.

### Morphological characterization of larval phenotype

Treated and control larvae (*N* = 10 per condition in each replicate) were anesthetized with Tricaine, mounted in 2% methylcellulose in H_2_O on depression slides, and imaged under a Leica M165 FC microscope equipped with a Nikon DS-Fi2 digital camera. Quantification of the main morphological traits (standard length, eye area and area of the swimming bladder) was performed using Fiji-ImageJ software (Version 2.14.0/1.54p). The experiment was repeated for 3 times.

### Lipids extraction for mass spectrometry analysis (LC–MS)

Heads and trunks of treated and control larvae (*N* = 30 in each condition) were separated with a sharp blade, pooled, snap frozen in liquid nitrogen, and stored at − 80 °C until use. Folch’s method [16] was used to extract lipids. Briefly, frozen pellets were resuspended with Milli-Q water and homogenized using a Pellet Pestle^®^ Motor (Kimble) for 30 s, alternating with 10 min incubation on ice (repeated for three times). After homogenization, a solvent solution composed of chloroform:methanol:water in a 8:4:3 volumetric ratio was added. The organic phase was collected, resuspended in the same solution and processed a second time to remove contaminants. Finally, samples were dried with a lyophilizer, flushed with nitrogen, sealed, and stored at − 80 °C. Subsequent LC–MS measurements were carried out to determine the internal concentration of BSSG in heads and trunks of treated larvae compared to controls. Lipid extracts were dissolved in 90:10 methanol/chloroform and 5 μL were injected into a Hewlett-Packard Model 1100 Series liquid chromatograph (Hewlett-Packard Development Company, CA) coupled to a photodiode array (PDA) detector (Agilent Technologies, Agilent 1100 Series) and to a Bruker Esquire-LC quadrupole ion-trap mass spectrometer (Bruker OptikGmbH) equipped with atmospheric pressure electrospray ion source. Quantitative LC–MS measurements were also performed in positive ion mode using a Triple Quadrupole (QQQ) mass spectrometer (Applied Biosystems, API 3000 QQQ) equipped with an electrospray ion source (ESI), and combined with a Shimadzu High Performance LC system (CBM-20 A, binary pump LC-20AB). Chromatographic analysis was carried out in both MS conditions at room temperature on a Kinetex-C8 100 × 4.6 mm, 2.6 μm column (Phenomenex, Italy). The eluent (1.0 mL/min) consisted of (A) methanol:water/10 mM ammonium acetate (70:30) and (B) methanol:isopropanol/10 mM ammonium acetate (90:10) using a linear gradient: 65%–100% B in 40 min, followed by isocratic B held for 10 min.

### Neutral red staining

Zebrafish larvae treated with either BSSG, β-sito or the control vehicle were incubated with Neutral red solution (553-24-2, Sigma-Aldrich) at a final concentration of 4 µg/mL for 3 h in the dark, and then briefly rinsed in Fish Water. Larvae were anesthetized with Tricaine and imaged under a Leica M165 FC microscope with a Nikon DS-Fi2 digital camera. The length of the stained portion of the mid-intestine was measured with Fiji-ImageJ. Each treatment was performed four times with at least 10 larvae per condition.

### Alcian blue staining in zebrafish larvae

Treated and control WT and *gr*^*−/−*^ zebrafish larvae were fixed overnight at 4 °C in 4% PFA (Sigma-Aldrich) in PBS. Staining with alcian blue (Alcian blue 8GX–A5268, Sigma-Aldrich Sigma) was performed as described in [17]. Number of goblet cells was manually scored starting from the junction between the intestinal bulb and the mid-intestine. The experiment was performed three times, with at least 10 larvae per condition.

### Neutrophil enumeration in Tg(mpx:GFP) and fluorescence analysis in Tg(NFkB:GFP), Tg(Stat3:EGFP) and *cyp11c1;*Tg(GRE:EGFP) larvae

Transgenic larvae were obtained crossing Tg(mpx:GFP)^i114^ [18], Tg(8xHs.NFκB:GFP, Luciferase)^hdb5^ (hereafter Tg(NFkB:GFP)) [19] or Tg(7xSRE-HSV.Ul23:EGFP)^ia28^ (hereafter Tg(Stat3:EGFP)) [20] x wild type zebrafish and *cyp11c1*^+/-^ x *cyp11c1*^+/-^ Tg(9xGCRE-HSV.Ul23:EGFP^ia20^) (hereafter Tg(GRE:EGFP)) [21]. At 3 dpf, larvae were screened for specific fluorescence and only GFP-positive individuals were randomly assigned to treated and control groups. After treatment, larvae were anesthetized and mounted in 1% low melting agarose. The same intestinal region, starting from the junction between the intestinal bulb and the mid-intestine and spanning at least 4 somites, was imaged by z-stacks (5 µm step size) under a 20× (for Tg(mpx*:GFP*), Tg(NFkB:GFP) and *cyp11c1*;Tg(GRE:EGFP)) or 40× objective (for Tg(7xStat3:EGFP)) on a Nikon C2 confocal microscope, using NIS ELEMENTS software. Fluorescent neutrophils in Tg(mpx:GFP) larvae were manually counted by scrolling through the z-stacks, assisted by the Cell counter tool in Fiji-ImageJ to avoid repeated counting, as described in [22]. Only neutrophils located in the intestinal walls along 5 somites were considered.

To quantify fluorescence signal in Tg(NFkB:GFP), Tg(Stat3:EGFP) and *cyp11c1*;Tg(GRE:EGFP) larvae, z-stack images were converted to maximum intensity z-projections, and mean fluorescence was measured in the same region of interest (ROI) using Fiji-ImageJ software. After the image acquisition, *cyp11c1*;Tg(GRE:EGFP) larvae were genotyped to distinguish *cyp11c1*^+/+^ and *cyp11c1*^*−/−*^;Tg(GRE:EGFP) siblings.

Each experiment was performed three times, with at least 10 larvae per condition.

### Fluorescence analysis of *cyp11c1;*Tg(GRE:EGFP) adults

*cyp11c1*^+/+^;Tg(GRE:EGFP) and *cyp11c1*^*−/−*^ Tg(GRE:EGFP) males of 4 mpf of age were randomly subdivided into treated (fed for 15 days with BSSG-enriched food) and control groups. At the end of the experimental period, fish were fasted 24 h prior to sacrifice to reduce the amount of food residues inside the intestine. After sacrifice, intestine was extracted and mounted in 1% low-melting agarose for imaging. Samples were imaged by z-stacks (7 µm step size) under a 20× objective on a Nikon C2 confocal microscope. To quantify intestine fluorescence, z-stack images were converted to maximum intensity z-projections, and the mean fluorescence was measured after setting a threshold to isolate the fluorescent region. Values were then normalized to the untreated *cyp11c1*^*−/−*^;Tg(GRE:EGFP), considered as the background signal.

### Acridine Orange staining in zebrafish larvae

Control and BSSG-treated 5-dpf zebrafish larvae were incubated with Acridine Orange solution (A6014, Sigma-Aldrich) at a final concentration of 15 µg/mL for 10 min in the dark, and then extensively rinsed in Fish Water. Larvae were then anesthetized and mounted in 1% low melting agarose. The same intestinal region was imaged as z-stacks (5 µm step size) under a 20× objective on a Nikon C2 confocal microscope, using the NIS ELEMENTS software. To quantify fluorescence, z-stack images were converted to maximum intensity z-projections, and the mean fluorescence intensity was measured in the same ROI spanning 4 somites with Fiji-ImageJ software.

### Peristalsis analysis

According to the protocol described in [8], control and BSSG-treated 5-dpf larvae were incubated with DCFH-DA (2’, 7’-Dichlorofluorescein diacetate, Sigma-Aldrich) at a final concentration of 1 mg/L overnight in the dark before the recording of peristaltic movements. Larvae were rinsed three times in Fish water, anesthetized for 1 min in Tricaine to limit its influence on muscular contraction, and mounted in 2% methylcellulose. Videos of 6–8 min were recorded under a Leica M165 FC fluorescence microscope. Each larva was analysed twice by scoring the number of peristaltic contractions, defined as partial or total invaginations of the intestinal bulb [23]. The average value was used to calculate the frequency of peristaltic movements over a 2 min period. The experiment was performed three times, with at least 10 larvae per condition.

### Gastrointestinal transit assay

According to the protocol described in [24] with slight modifications, gastrointestinal transit was evaluated as the progression of the food bolus along the digestive tract over 24 h after feeding. Treated and control 5-dpf larvae were fed commercial food in the morning (8:00 a.m.) and allowed to feed for 2 h. This was considered as “Time 0”. Larvae showing the intestinal bulb filled with a visible food bolus under brightfield were considered for the analysis and imaged under a Leica M165 FC microscope 4, 8 and 24 h post-feeding. Food progression was then evaluated according to the scoring system described in [24]. A total of *N* = 32 CTR and *N* = 24 treated larvae were examined.

### Ex vivo analysis of zebrafish gut contractility

The intestinal contractility of adult zebrafish was analysed ex vivo by measuring tension changes with the isolated organ bath technique, as previously described in mouse [25, 26] and fish preparations [27]. Experiments were performed on the full-length intestine of zebrafish fasted for 24 h prior to sacrifice to reduce the amount of food residues in the lumen. Once extracted, the intestine was maintained in Krebs solution (NaCl 118 mM, KCl 4.7 mM, CaCl_2_∙2H_2_O 2.5 mM, MgSO_4_∙7H_2_O 1.2 mM, K_2_HPO_4_ 1.2 mM, NaHCO_3_ 25 mM, C_6_H_12_O_6_ 11 mM). By creating two loops with silk thread, each intestine was mounted along its longitudinal axis in an organ bath containing 10 mL of oxygenated (95% O_2_ + 5% CO_2_) Krebs solution maintained at 28.5 °C. Changes in muscle tension were recorded by isometric force transducers (World Precision Instruments, Berlin, Germany) connected to a PowerLab 4/30 data acquisition system using LabChart 8 software (ADInstruments, Besozzo, VA, Italy). Intestinal preparations were stretched to an initial tension of 0.1 g and left to equilibrate for 45 min to allow the development of rhythmic spontaneous contractions [25, 26]. At the end of the equilibration period, samples were activated using 1 μM carbachol (CCh), a non-selective cholinergic receptor agonist, directly added to the Krebs solution. To evaluate smooth muscle contraction, each intestine was exposed to 40 mM KCl, a depolarizing agent that induces Ca^2+^ release and subsequent smooth muscle contraction. To assess the cholinergic receptor-mediated excitatory responses, samples were then exposed to increasing concentrations of CCh (0.001–100 μM) added cumulatively to the organ baths to generate concentration–effect curves. Neuronal-mediated contractions were evaluated using electrical field stimulation (EFS) at increasing frequencies with constant voltage (0–40 Hz; 1ms pulse duration; 10s pulse-trains, 80 V) via platinum electrodes connected to an S88 stimulator (Grass Instrument), inducing membrane depolarization and neurotransmitter release. Finally, to characterize the inhibitory neuromuscular responses, zebrafish intestines were exposed to 0.1 μM isoprenaline, a non-selective β-adrenergic receptor agonist. Concentration–response curves were subjected to a nonlinear regression analysis (fitted to a sigmoidal equation) to calculate maximal tension (Emax) values. Contractile responses were expressed as gram tension/gram dry tissue weight [25, 26]. *N* ≥ 4 animals were analysed.

### Microbiota sequencing

#### Zebrafish gut microbiota sequencing

Adult zebrafish treated for 30 days with BSSG-enriched food and their respective controls were sacrificed after 24 h of fasting, to allow gut clearance of food residues. The whole intestine was extracted, snap-frozen in liquid nitrogen, and stored at − 80 °C until used. Bacterial DNA extraction was performed with the DNeasy^®^ PowerSoil^®^ Pro Kit (Qiagen), according to the manufacturer’s instructions, with slight modification: to ensure efficient disruption of bacterial cell walls, samples were repeatedly homogenized using a BeadBug™ microtube homogenizer (Merck Life Sciences) and then continuously vortexed for 20 min until complete tissue destruction. Subsequent steps followed the Kit protocol. DNA samples were quantified with a Qubit fluorometer (ThermoFisher) and diluted to 10 ng/µl. Bacterial DNA was then amplificated with a nested PCR protocol to obtain 16S rRNA gene sequences. In the first PCR reaction (PCR I), DNA was amplified with forward Eub8F and reverse 984yR primers with Platinum TM Taq DNA Polymerase High Fidelity (ThermoFisher). 1 µl of this PCR I product was used as template for the second PCR (PCR II), using internal primers 515 F and 806R to amplify the V4 hypervariable region of the bacterial 16S rRNA gene, which allows to discriminate the different bacterial taxa [28]. PCR conditions were as follows: 94 °C for 1 min to activate the DNA polymerase, 25 cycles of 94 °C for 30 s, 53 °C (in PCR I) or 57 °C (in PCR II) for 30 s, and 68 °C for 45 s, followed by a final extension at 68 °C for 7 min. Primer sequences are listed in Additional file 2: Table 2. The sequencing of the 16S region was performed exploiting the Illumina sequencing platform to obtain pair-end sequences of 150 bp. Reads were processed to assign Amplicon Sequence Variants (ASVs) to the bacterial taxa using the pipeline described in [29]. For these analyses, 3 WT and 3 *gr*^*−/−*^ male controls were compared with 4 WT and 4 *gr*^*−/−*^ treated male zebrafish.

#### Mouse stool microbiota sequencing

Bacterial DNA was extracted from fecal samples using QIAamp Fast DNA Stool Mini Kit (51604, Qiagen) using the pathogen detection protocol, optimized to maximize the ratio of non-mouse DNA. DNA was subsequently amplified with Phusion® High-Fidelity DNA Polymerase (BioLabs) using forward 515 F and reverse 806R primers (Additional file 2: Table 2) as follows: 98 °C for 3 min followed by 25 cycles of 95 °C for 45 s, 58 °C for 45 s and 72 °C for 50 s, followed by a final extension at 72 °C for 5 min. The sequencing of the 16S region was performed by the sequencing facility of Biology Department at the University of Padova exploiting the Illumina sequencing platform to obtain pair-end sequences of 150 bp. Amplicon Sequence Variants (ASVs) were then assigned to the bacterial taxa and mean relative abundance at phylum and family level were calculated. Data were obtained from *N* = 2 male mice/condition.

### RNA extraction, reverse transcription and Real Time-quantitative PCR (RT-qPCR)

Total RNA was extracted with TRIzol reagent (15596026, Thermo Fisher Scientific) from different tissue samples: pooled whole larvae at 5 dpf (at least *N* = 15 in each sample); pooled whole larvae at 30 dpf (*N* = 14 in each sample); single adult zebrafish intestine or brain; and single mouse small intestine samples approximately 1.5 cm in length. The quantity and quality of the isolated RNA were assessed using a NanoDrop 2000c (Thermo Fisher Scientific) and TapeStation System 4150 (Agilent). 1 µg (for zebrafish larvae and mouse tissue samples) or 500 ng (for adult zebrafish tissue samples) of total RNA were treated with RQ1 RNase-Free DNase (M6101, Promega) and used for cDNA synthesis, with the High-Capacity cDNA Reverse Transcription Kit (4368814, ThermoFisher). cDNA was diluted 1:10 (or 1:5 for mouse intestine samples) and amplified using 5× HOT FIREPol EvaGreen qPCR Mix Plus (08–36-00001, Solis BioDyne) with locus-specific primers. Genes expression was measured with SybrGreen method on a CFX384 Touch Real-Time PCR Detection System (BioRad). Primers’ melting curves and threshold cycles (Ct) were automatically generated. Relative gene expression levels were calculated using the comparative Ct method (2^–ΔΔCt^) normalized to housekeeping genes (*β-actin* for adult zebrafish tissues, *ef1α* for larval zebrafish samples; *Tbp* for mouse intestine samples). Primers used for RT-qPCR analysis are listed in Additional file 2: Table 3.

### RNA sequencing (RNAseq)

Library construction from RNA extracted from 30 dpf zebrafish larvae was performed using the QuantSeq 3ʹ mRNASeq Library Prep Kit for Illumina (015UG009V0260, Lexogen) according to manufacturer’s protocol. Briefly, 500 ng of total RNA were retrotranscribed with oligod(T) primers and, after RNA degradation, the second cDNA strand was synthesized using random primers. Double-stranded DNA was amplified to incorporate sequencing adapter and library barcodes. For mouse samples, libraries were constructed starting from at least 500 ng of total RNA following ribosomal RNA depletion, using Illumina Ribo-Zero Plus rRNA Depletion Kit (Illumina). rRNA-depleted RNA was subsequently purified by ethanol precipitation and employed for library preparation. Briefly, after fragmentation, the first-strand cDNA was synthesized using random hexamer primers (NEBNext Ultra II RNA First Strand RNA Synthesis Module; NEB). Then, the second strand cDNA was synthesized, and dUTPs were replaced with dTTPs in the reaction buffer (NEBNext Ultra II Directional RNA Second Strand Synthesis Module; NEB). Double-strand DNA was purified using magnetic beads, end-repaired, and A-tailed to facilitate adapter ligation. After gel size selection (250–800 bp), USER enzyme digestion (NEB) was employed to eliminate UTP-containing second strand cDNA filaments. Prior to sequencing, libraries were quantified using the Qbit (dsDNA Quantitation, High Sensitivity; Thermofisher) and size distribution was assessed using the High Sensitivity DNA kit (Agilent Technologies). Zebrafish libraries were sequenced by the Sequencing facility of the Department of Biology, University of Padova, using a NextSeq 500 (Illumina) platform, employing a single read approach. Conversely, mouse libraries were sequenced by Novogene facility using Novoseq X plus (Illumina) sequencer employing a paired-end approach.

### Transcriptome data analysis

After adapter trimming with Cutadapt (version 4.7) [30], transcript expression was quantified using Salmon method (version 1.10.3) [31]. The resulting count matrix was imported into the R statistical environment, and the edgeR package (version 4.0.2) [32], was used for gene expression normalization and to identify differentially expressed genes (DEGs) between treated and control samples (FDR ≤ 0.05), reported in Additional file 4 and Additional file 5. Zebrafish and murine DEGs were used for Gene Ontology (GO) enrichment analysis via *enrichplot* package in R software, and bar charts were generated on ShinyGO [33]. Raw RNAseq data have been deposited in the SRA database.

### Electron microscopy and ultrastructural analysis of mouse microvilli

After sacrifice, mouse small intestine was extracted and gently flushed with cold PBS. Small sections (0.5 cm long) of mouse small intestine were fixed overnight at 4 °C with Karnovsky fixative (2.5% glutaraldehyde, 2% paraformaldehyde in 0.1 M cacodylate buffer), washed with 0.1 M cacodylate buffer, post-fixed with osmium tetroxide for 2 h, and embedded in EMbed 812 (Electron Microscopy Sciences). Ultrathin sections were stained with uranyl acetate and lead citrate and examined using a Philips M400 microscope operating at 100 kV. The microvilli length was measured from the tip to the base with Fiji-ImageJ software. Analyses were repeated on multiple images derived from 3 individuals per condition.

### Alcian blue staining of mouse small intestine histological sections

After sacrifice, mice small intestine was extracted and gently flushed with cold PBS. Segments approximately 1.5 cm in length were fixed in Bouin fixative solution (30 mL saturated picric acid in ddH_2_O, 10 mL formaldehyde, 2 mL glacial acetic acid for 42 mL) for 24 h and then rinsed in 70% ethanol. Samples were dehydrated in graded ethanol (80%−90%−100% in ddH2O) for 1 h at RT, cleared in xylene (Sigma-Aldrich), infiltrated with xylene: paraffin (1:1) and embedded in paraffin. Sections (7 µm) were cut using a Leica microtome. Tissues sections were deparaffinized, rehydrated to ddH_2_O, incubated in 3% glacial acetic acid solution for 3 min, and stained with Alcian blue solution (1% Alcian blue 8GX – A5268, Sigma-Aldrich in 3% glacial acetic acid; pH 2.5) for 30 min. Sections were rapidly rinsed in 3% glacial acetic acid solution, washed in running tap water for 5 min, and counterstained with Eosin Y (Sigma-Aldrich, USA). Then were dehydrated in 100% ethanol and mounted for visualization. Alcian blue-stained goblet cells were counted along intestinal villi in 3 sections spaced 100 µm in 6 mice for each condition.

### Macrophages staining on mouse small intestine histological sections

Mouse small intestines were paraffin-embedded and sectioned as previously described and mounted on Superfrost® Plus microscope slides (J1800AMNZ, Thermo Scientific). Sections were deparaffinized, rehydrated to ddH_2_O with graded ethanol series (100%−90%−70% in ddH2O) for 5 min each and incubated for 15 min in a quenching solution (NH_4_Cl 50 mM) to reduce autofluorescence. Antigen retrieval was performed by incubation in sub-boiling citrate buffer (citric acid 0.01 M, pH 6) for 10 min, followed by 10 min in TBS-1% Tween at RT. Slides were then blocked for 1 h in saturating solution (15% goat serum, 2% BSA, 0.25% gelatine, 0.2% glycine in PBS supplemented with 0.5% Triton X-100). Sections were incubated overnight at 4 °C in F4/80 monoclonal primary antibody (14–4801-82, Thermofisher Scientific) diluted 1:50. After 3 washes for 5 min in TBS-1% Tween, sections were incubated for 1 h at RT with goat anti-rat-Alexa Fluor 568 secondary antibody (A-11077, Thermofisher Scientific) at a final dilution of 1:200. To reduce autofluorescence, slides were treated with Sudan Black solution (0.1% in EtOH 70%) for 10 min, followed by extensive washing in TBS-1% Tween. Nuclei were stained with DAPI for 5 min and sections were mounted with SlowFade™ Diamond Antifade Mountant (S36967, Thermofisher Scientific). Images were acquired using a Zeiss AXIO Zoom.V16 fluorescence microscope equipped with an Axiocam 305 mono camera. Macrophages were manually counted in sections from 3 mice per condition.

### Radioligand binding assay

To verify the ability of BSSG to bind steroid hormone receptors at a concentration of 10 µM, a radio-ligand binding assay was performed by Eurofins Panlabs Discovery Services using the NHR Binding Agonist Radioligand Assay. BSSG selectivity for human androgen receptor, estrogen receptor, glucocorticoid receptor, mineralocorticoid receptor and progesterone receptor were calculated as the percentage of inhibition for the binding of a radio-labeled ligand, specific for each receptor ([^3^H]-methyltrienolone, [^3^H]-estradiol, [^3^H]-dexamethasone, [^3^H]-aldosterone and [^3^H]-progesterone, respectively).

### Zebrafish whole-mount immunofluorescence

After BSSG treatment, 5-dpf larvae were fixed overnight in 4% PFA (Sigma-Aldrich) in PBS at 4 °C and dehydrated in 100% methanol. After rehydration with graded methanol series (75–50–25% methanol in PBS) and PBT (0.2% Triton X-100 in PBS) for 5 min each at RT, larvae were depigmented (2% KOH and 3% H_2_O_2_ in PBT for 5 min, followed by a 5 min wash in ddH_2_O) and permeabilized (15 min at − 20 °C in ice-cold 100% acetone, followed by washes in ddH_2_O and PBT). After a step in a blocking solution (1% BSA and 5% sheep serum in PBT) for 4 h at RT, larvae were incubated for 48 h at 4 °C with pan-neuronal anti-HuC/D (A-21272, ThermoFisher) or anti-Sox10 (GTX128374-S, GeneTex) primary antibody diluted 1:200 in the blocking solution. Larvae were then washed four times in PBT for 20 min each at RT, saturated in the same blocking solution for 4 h and incubated overnight in the dark at 4 °C with Streptavidin conjugate Alexa Fluor 555 secondary antibody (S32355, ThermoFisher) against HuC/D or goat anti-rabbit-Alexa Fluor 488 (A11034, Invitrogen) secondary antibody against Sox10 diluted 1:1000. After extensive washes in PBT, larvae were mounted in 1% low-melting agarose and imaged by z-stacks (3 µm step size) under a 20× objective at Nikon C2 confocal microscope. The same body region was imaged for all larvae. All HuC/D^+^ enteric neurons and Sox10^+^ neuronal progenitors visible on the ventral side of the intestine were manually scored by scrolling through the z-stacks, assisted by the Cell counter tool in Fiji-ImageJ to avoid repeated counting. *N* ≥ 10 animals/condition. The experiment was repeated three times.

### Fish embryo acute toxicity test

FET test was performed according to the Organization for Economic Co-operation and Development (OECD, Paris, France) Guideline No. 236 (2013) [34] to define a non-lethal and non-toxic BSSG concentration for zebrafish treatment, in order to avoid teratogenic or developmental issues.

Briefly, 6 hpf embryos were transferred singularly into 24 well plates (1 embryo in 1 mL solution/well) and incubated with increasing concentrations of BSSG (2.5–5–10–20–40 µM) or the corresponding solvent control (DMSO at 0.1%–0.2%–0.4%). For each concentration, 20 embryos were individually incubated with BSSG, and the remaining 4 wells were used as internal negative controls (Fish Water). Negative and positive controls (1.5% EtOH) were also tested. The embryo medium was changed daily, and the developmental status of zebrafish embryos and larvae was monitored until 4 dpf. Survival and hatching rates, presence of cardiac edema and of swimming bladder were assessed based on the total number of surviving embryos.

### Statistical analysis

Statistical analyses were performed using Graph Pad Prism V10.2.3. Data are expressed as mean ± SEM and statistical significance was calculated using unpaired Student's *t*-test for comparisons between two groups or one-way ANOVA followed by Tukey’s post-hoc test for multiple comparisons. For ex vivo gut contractility analyses, two-way ANOVA followed by Bonferroni post hoc test was used for multiple comparison. The differences between groups were considered significant at *P* < 0.05. Post hoc tests were run only if F achieved *P* < 0.05, and there was no significant variance inhomogeneity. For the gastrointestinal transit assay, differences in the distribution of food bolus across transit zones between control and treated larvae were assessed at each time point using Fisher’s exact test for 2 × 5 contingency tables based on absolute larval counts. *p*-values are indicated with the following symbols: *P < 0.05; **P < 0.01; ***P < 0.001; ****P < 0.0001; ns, not significant.

## Results

### BSSG administration triggers intestinal inflammation in zebrafish larvae

Zebrafish larvae exposed to 10 µM BSSG presented normal development (Additional file 3: Figure 1 A, B) and mass spectrometry analysis of lipids extracted from larval heads and trunks confirmed the accumulation of this compound in the trunk region of treated animals (Additional file 3: Figure 1 C).

**Fig. 1:**
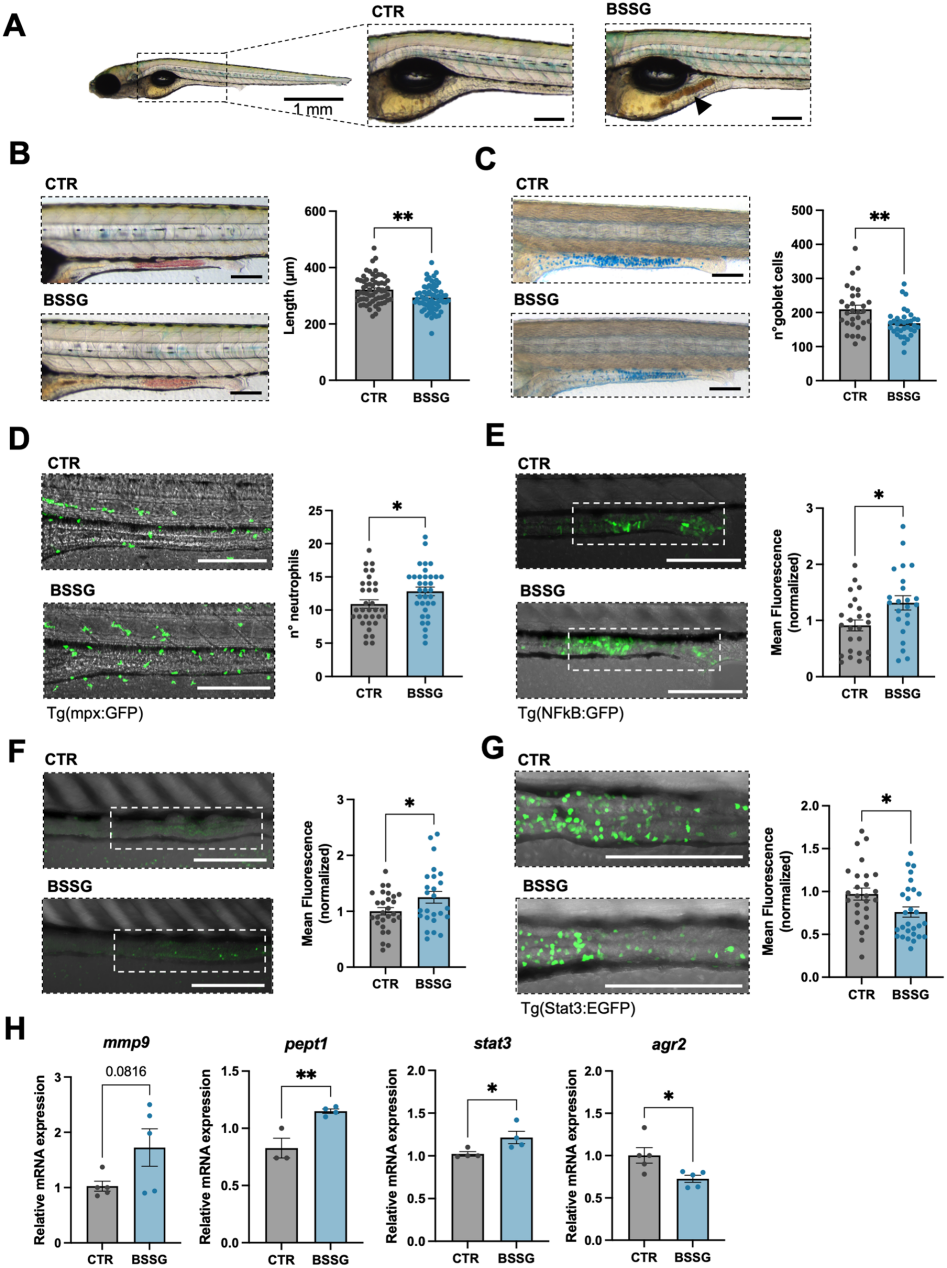
Evidence of intestinal inflammation in zebrafish larvae. **A** Representative intestine morphology of 5-dpf zebrafish larva and corresponding magnification of CTR (left) and BSSG-treated (right). Arrowhead indicates dark clumps in the mid-intestine of a BSSG-treated larva. **B** Analysis of the mid-intestine region stained with neutral red and quantification of its length. **C** Analysis of goblet cells number stained with alcian blue in the mid-intestine of CTR and BSSG-treated larvae. **D** Representative micrographs of the distribution and number of fluorescent neutrophils in the mid-intestine of CTR and BSSG-treated Tg(mpx:GFP) larvae. **E** Analysis of fluorescent signal in the mid-intestine of Tg(NFkB:GFP) CTR and BSSG-treated larvae. **F** Analysis of fluorescent signal in Acridine Orange-stained CTR and BSSG-treated larvae. **G** Representative micrographs of the distribution and number of fluorescent stem-like cells labelled by Stat3 expression in the mid-intestine of CTR and BSSG-treated Tg(Stat3:EGFP) larvae. Dotted lines evidence the analysed regions. **H** RT-qPCR analysis of inflammation-related genes in whole tissue of pooled CTR and BSSG-treated larvae in N ≥ 3 biological replicates. Data are expressed as mean ± SEM. Statistical analysis was performed using unpaired Student’s t-test: * *P* < 0.05; **P < 0.01. Scale bar: 200 µm

The presence of dark aggregates in the intestine of nearly all larvae exposed to BSSG prompted us to hypothesize that the gut may represent its first target (Fig. 1A). Consistently, in vivo analysis of acidified lysosomes evidenced a reduction in the number of lysosome-rich enterocytes (LREs) in treated larvae (Fig. 1B). To determine whether these intestinal effects were specifically attributable to BSSG rather than to possible metabolites, we performed the same assay on larvae treated with β-sitosterol, which shares the chemical structure of BSSG but lacks the glucose moiety. This molecule did not affect LREs (Additional file 3: Figure 2 A), suggesting that the detrimental effects only depend on BSSG consumption.

Mucus-secreting goblet cells along the mid-intestine, essential for protecting the gut against digestive enzymes and external insults, were reduced after exposure to BSSG (Fig. 1C). Conversely, the number of neutrophils infiltrating the mid-intestine was markedly increased in treated larvae (Fig. 1D), providing an indication of intestinal inflammation onset. This was further supported by increased fluorescence in the intestine of BSSG-treated transgenic Tg(NFκB:GFP) larvae, indicating the activation of NF-κB pathway (Fig. 1E). Consistently with the occurrence of an inflammatory phenotype, we also observed increased apoptosis in the mid-intestine (Fig. 1F).

Moreover, the reduction of Stat3-linked fluorescence specific of stem-like cells located at the base of the intestinal folds [20], which are analogous to crypt base columnar cells in mammals, may reflect a depletion of proliferating cells (Fig. 1G).

To further characterize the impact of BSSG on gut homeostasis, we analysed the expression of inflammation-related markers in whole larvae following acute exposure. We observed a trend towards increased expression of *mmp9* (*matrix metallopeptidase 9*), along with significant upregulation of *stat3* (*signal transducer and activator of transcription 3*) and *pept1* (*peptide transporter 1*). Reduced expression of *agr2* (*anterior gradient 2*), involved in mucus production by goblet cells, further supported the presence of a defective intestinal epithelial barrier (Fig. 1H).

We also observed reduced expression levels of the autophagy-related genes *atg5* (*autophagy*  *related* *5*) and *lc3b* (*microtubule associated protein 1 light chain 3b*), suggesting a possible impairment of autophagy (Additional file 3: Figure 2B).

Taken together, these results depict a scenario in which BSSG administration induces intestinal inflammation, disrupting essential cellular processes, potentially exacerbating gut dyshomeostasis.

### BSSG treatment alters gut motility and microbial composition

**Fig. 2:**
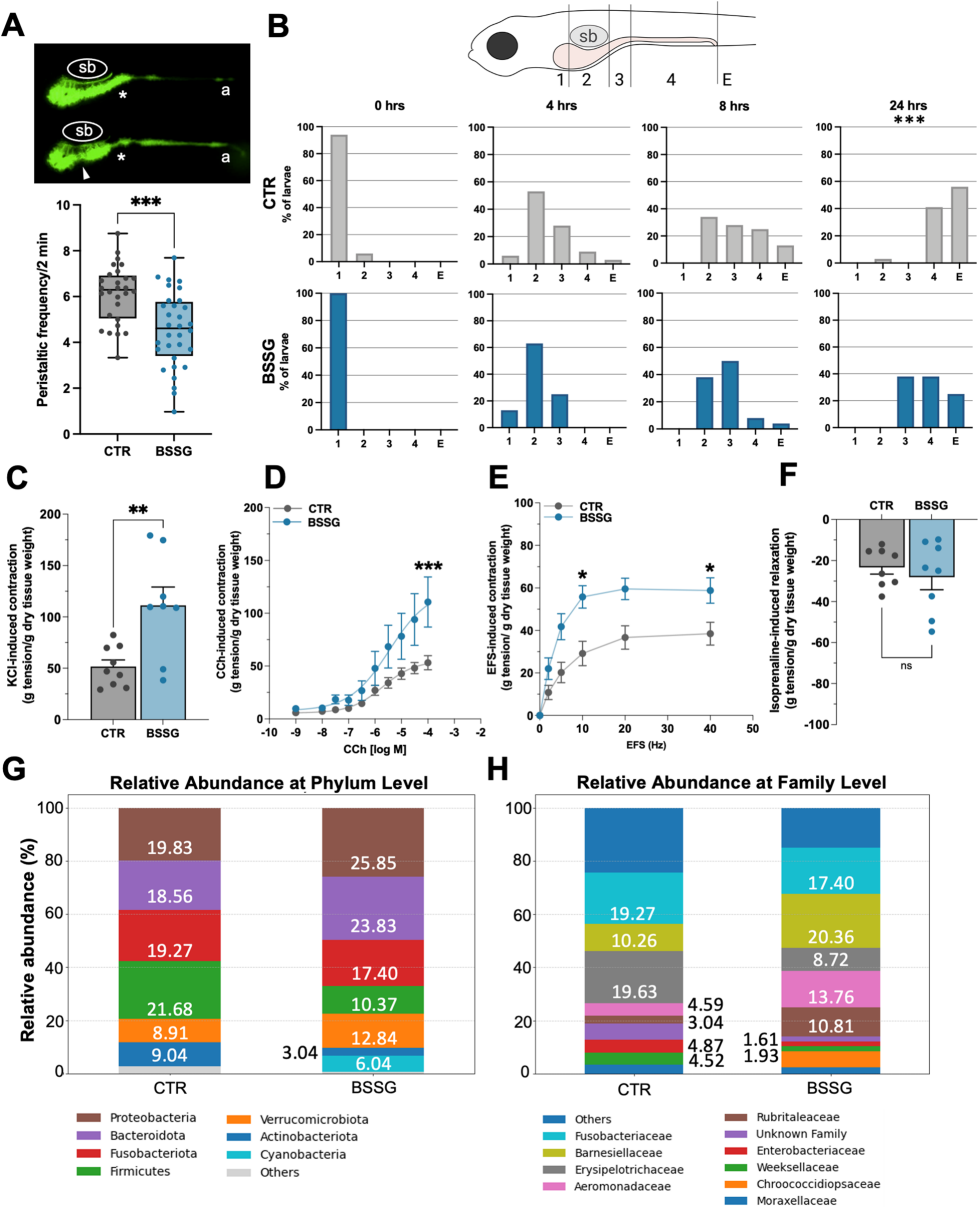
Impairment of intestinal motility and changes in gut microbiota. **A** Upper panel: video frames showing intestinal peristalsis. Asterisk evidences the point where muscle contraction originates. Arrowhead indicates intestinal bulb invagination. sb, swimming bladder; a, anus. Lower panel: quantification of intestinal peristaltic movements scored from live imaging videos and then calculated as a frequency every 2 min. Data were generated from N = 3 biological replicates. Box and whiskers graph shows the median and the min/max values. **B** Upper scheme: regions subdividing zebrafish larval intestine defined as “Zones”. 1: portion of the intestinal bulb rostral to the sb; 2: intestinal bulb below the sb; 3: junction between the intestinal bulb and the mid-intestine; 4: mid- and posterior intestine. When the intestine is empty, it is referred as E. Lower panel: bar graphs represent the profile of gastrointestinal transit reported as percentage of larvae that show the most rostral extent of the food bolus in one of the defined zones at different time points, based on visual analysis of alive individuals. Statistical significance refers to global differences assessed using Fisher’s exact test on absolute counts. ***P < 0.001. N = 32 CTR and 24 BSSG-treated larvae deriving from different spawns. **C–F** Ex vivo analysis of intestinal neuromuscular contraction elicited by: 40 mM KCl (**C**); concentration–response curves to CCh (0.001—100 μM) (**D**); electric field stimulation (EFS; 0–40 Hz, 80 V) (**E**); intestinal relaxation to 0.1 μM isoprenaline (**F**), of isolated intestinal preparations of adult zebrafish with or without in vivo BSSG treatment. Each dot represents a tissue sample from a single adult individual. N ≥ 8 animals/condition. Data are expressed as mean ± SEM. Statistical analysis was performed using unpaired Student’s t-test for two-sample comparisons, or a two-way ANOVA followed by a Bonferroni post hoc test for multiple comparisons; *P < 0.05; **P < 0.01; ***P < 0.001; ns, not significant **G, H** Analysis of adult zebrafish gut microbiota. The bar graphs show the percentage of the relative abundance in bacterial phyla (**G**) and families (**H**)

To study the impact of BSSG on intestinal physiology, we firstly analysed peristaltic frequency. Treated larvae exhibited a lower number of gut contractile waves compared to controls (Fig. 2A). To corroborate this finding, we monitored larval gastrointestinal transit along the digestive tract, ideally divided into transit zones [24] (Fig. 2B, upper panel). Over time, differences in food bolus localization indicated that treated larvae displayed delayed gastrointestinal transit (***P < 0.001 with Fisher’s exact test 24 h after feeding) (Fig. 2B, lower panel).

Since peristaltic activity is regulated by the enteric nervous system (ENS), we evaluated the number of differentiated enteric neurons and neuronal precursors (labelled with HuC/D and Sox10, respectively), but found no differences between treated and control larvae (Additional file 3: Figure 2 C), suggesting that BSSG may affect the functionality of enteric innervation rather than its density.

Therefore, we investigated the involvement of enteric neurotransmission in gut dysmotility in adult zebrafish fed with a BSSG-enriched diet over a prolonged period, to mimic chronic exposure. For the first time, both receptor-mediated and non-receptor-mediated neuromuscular responses of isolated whole intestines were evaluated in zebrafish. Muscular contractile responses were analysed by exposing zebrafish intestine to KCl, a depolarizing agent that induces Ca^2+^ release and subsequent smooth muscle contraction. A significant increase in the KCl-induced contraction was observed in intestinal preparations of treated individuals (Fig. 2C). To further evaluate the excitatory cholinergic response, gut samples were exposed to increasing concentrations of carbachol (CCh), a non-selective cholinergic agonist. Cumulative concentration–response curves revealed a significant increase in CCh-mediated contraction in treated intestines (Fig. 2D), suggesting altered cholinergic neurotransmission following BSSG treatment. Then, to verify the impact on ENS neuronal activity, intestinal preparations were subjected to electrical field stimulation (EFS) at increasing frequencies with constant voltage, to induce neuronal depolarization and consequent neurotransmitters release. Treated individuals displayed an increased excitatory neuromuscular response, determining a significant upward shift of the frequency–response curve to EFS (Fig. 2E). Higher 10 Hz-EFS-mediated contraction confirmed alterations in excitatory cholinergic response. Conversely, muscular relaxation induced by isoprenaline, a non-selective β-adrenergic receptor agonist, was not affected by treatment (Fig. 2F).

Next, given the relevance of the microbiota in maintaining intestinal homeostasis and the well-established association between its alterations and intestinal inflammation in humans and animal models [35], we analysed microbiota composition in adult zebrafish following BSSG exposure. At the phylum level, *Proteobacteria*, *Bacteroidota* and *Verrucomicrobiota* increased, while *Firmicutes* and *Actinobacteriota* decreased (Fig. 2G), resulting in a reduced *Firmicutes/Bacteroidetes* ratio, a widely recognized indicator of gut health [36]. At the family level, we observed an increase in *Barnesiellaceae*, *Aeromonadaceae* and *Rubritaleaceae,* and a reduction in the relative abundance of *Erysipelotrichaceae, Enterobacteriaceae* and *Weeksellaceae* (Fig. 2H). Overall, although these results did not reach statistical significance, likely due to the limited sample size, they suggest that BSSG treatment may influence the gut microbial composition, potentially contributing to the onset of dysbiosis.

### Transcriptome analysis of chronically treated larvae reveals intestinal dyshomeostasis, possibly prodromal to neurodegeneration

**Fig. 3:**
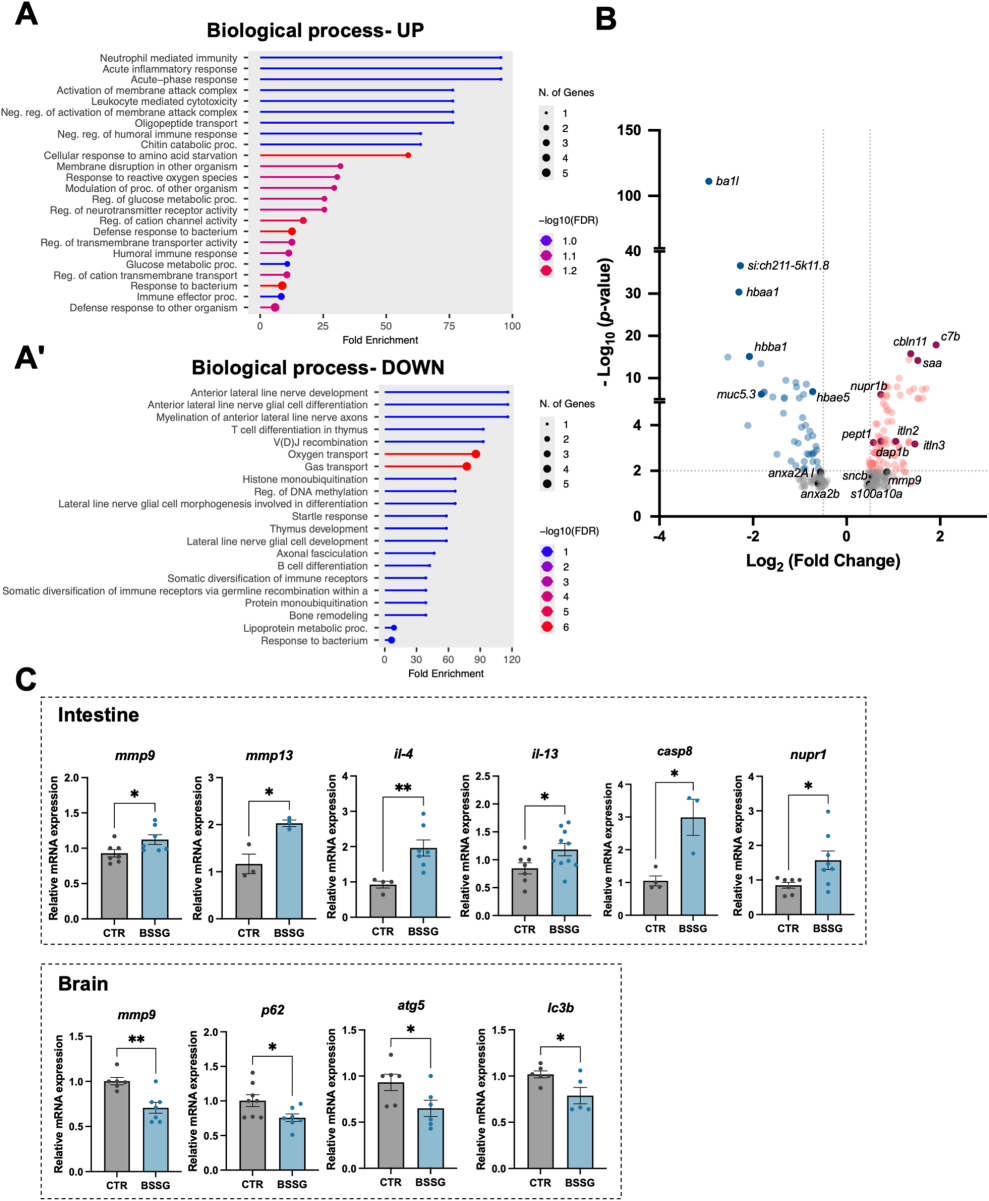
Zebrafish transcriptome analysis. **A** Bar charts of RNAseq analysis of RNA samples from pooled 30 dpf chronically treated whole zebrafish larvae. GO enrichment for the upregulated genes (GO Biological process). **Aʹ** GO enrichment for the downregulated genes (GO Biological process). **B** Volcano plot of differentially expressed genes in chronically treated larvae. Blue tones refer to downregulated DEGs, red tones to upregulated DEGs. Grey tones indicate not significant DEGs below the statistical threshold of *P* < 0.01. Log2(Fold Change) = ± 0.5 are indicated with a dotted line. **C** RT-qPCR analysis of different inflammation-, cellular stress- and autophagy-related genes in RNA samples from adult zebrafish intestines (upper panel) and brains (lower panel). Each dot represents a tissue sample from a single individual. N ≥ 3 animals/condition. Data are expressed as mean ± SEM. Statistical analysis was performed using unpaired Student’s t-test. *P < 0.05; **P < 0.01

RNAseq analysis of chronically treated zebrafish larvae revealed 261 differentially expressed genes (168 upregulated and 93 downregulated) compared to controls. Upregulated genes were mainly involved in *acute inflammatory response* as well as in *response to reactive oxygen species* and *defence response to bacterium* (Fig. 3A). The upregulation of both *mmp9* and *pept1*, in association with increased expression of *saa* (*serum amyloid a*) and *s100a10a* (*s100 calcium binding protein a10 a*) (Fig. 3B), corroborated previous findings in treated larvae (Fig. 1H), indicating the presence of gut inflammation.

BSSG treatment downregulated genes mainly associated with *lateral line nerve development* and *oxygen transport* (Fig. 3Aʹ). Decrease of *oxygen binding* resulted also among downregulated molecular functions (Additional file 3: Figure 3 A, right panel). Notably, the entire group of hemoglobin (*hb*) genes exhibited a significant reduction in treated individuals (Fig. 3B; Additional file 3: Figure 3B), a finding recently associated with the pathophysiology of different neurodegenerative diseases [37–39]. The decreased expression of *mucin 5.3* (*muc5.3*) (Fig. 3B), secreted by goblet cells to neutralize digestive enzymes and pathogens, is consistent with the reduced goblet cells number observed in treated larvae (Fig. 1C).

To validate transcriptome results, we tested the expression of specific genes in the intestine of adult zebrafish confirming the upregulation of inflammation-related markers (*mmp9, mmp13*, *il-4*, *il-13*) and cellular stress markers (*casp8*, *caspase 8*; *nupr1*, *nuclear protein 1*) (Fig. 3C, upper panel).

Since RNAseq analysis evidenced the alteration of genes potentially involved in neurodegeneration, we also evaluated adult zebrafish brains. In contrast to intestinal findings, *mmp9* expression in the brain was downregulated following treatment, potentially impacting its role in central nervous system (CNS) plasticity. We also observed the downmodulation of autophagy-related genes *atg5*, *lc3b* and *p62* (Fig. 3C, lower panel).

### BSSG-fed mice exhibit reduced weight and hallmarks of intestinal inflammation

**Fig. 4:**
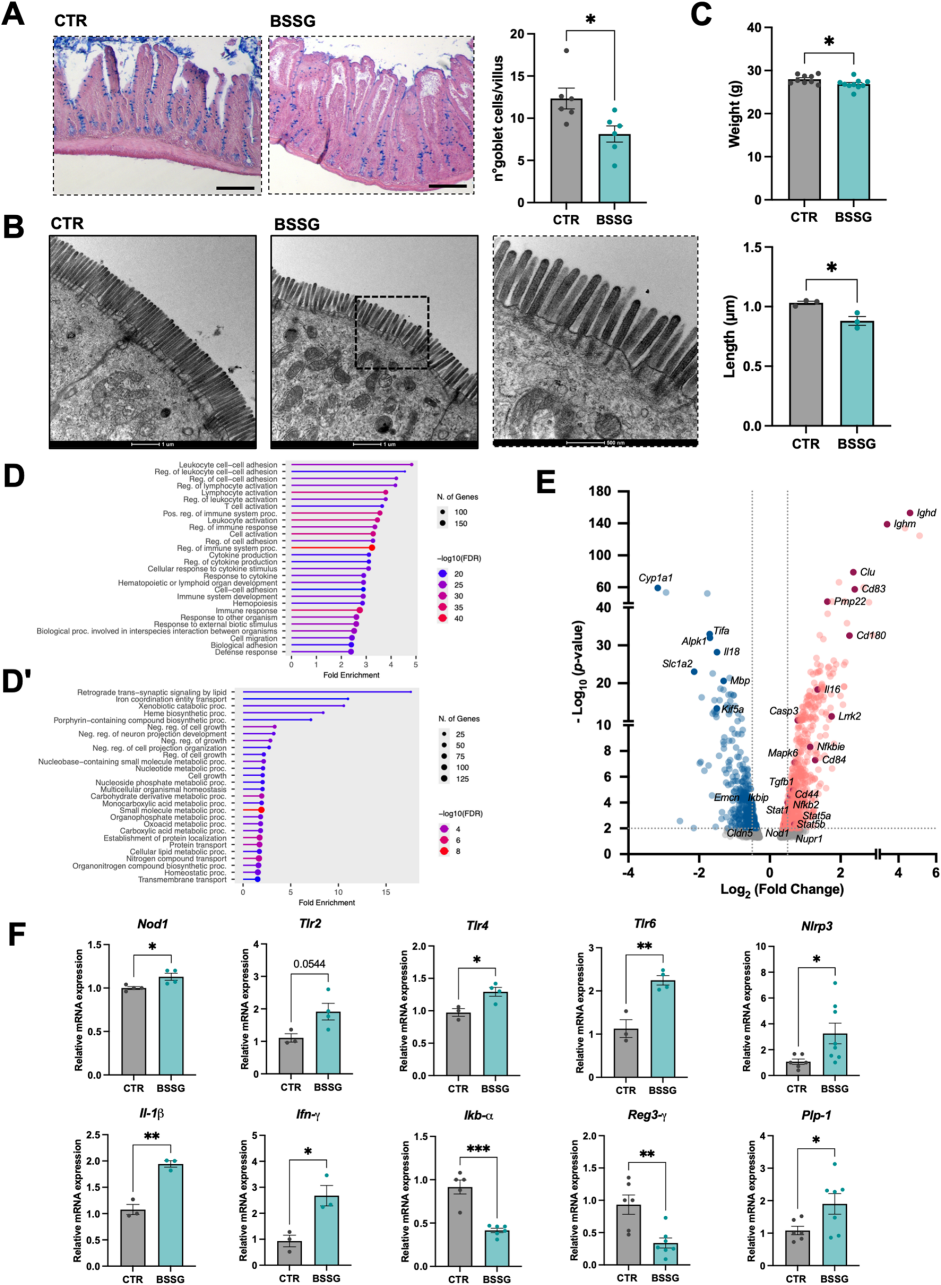
Evidence of gut inflammation in mouse model and intestinal transcriptome analysis. **A** Magnification of mouse small intestine histological sections stained with alcian blue and quantification of goblet cells number (3 images from N = 6 animals/condition). Scale bar: 200 µm. **B** Transmission electron microscopy of microvilli at the apical surface of mouse enterocytes. Scale bar: 1 µm. In the magnification box, microvilli of a treated mouse. Scale bar: 500 nm. Quantification of microvilli length. N = 3 animals/condition. **C** Mice body weight after 15 weeks of BSSG-enriched food diet. **D** Bar charts of RNAseq analysis of RNA samples extracted from mouse small intestine. GO enrichment of upregulated genes (GO Biological process). **Dʹ** GO enrichment of downregulated genes (GO Biological process). **E** Volcano plot of differentially expressed genes. Blue tones refer to downregulated DEGs, red tones to upregulated DEGs. Grey tones indicate not significant DEGs below the statistical threshold of *P* < 0.01. Log2(Fold Change) = ± 0.5 are indicated with a dotted line. **F** RT-qPCR analysis of different inflammation-related genes in RNA samples from mouse small intestine. Each dot represents a tissue sample from a single individual. Bar graphs show the mean ± SEM. N ≥ 3 animals/condition. Statistical analysis was performed using unpaired Student’s t-test. *P < 0.05; **P < 0.01; ***P < 0.001

To investigate whether BSSG dietary consumption could determine intestinal alterations also in the mouse model, WT mice were fed a BSSG-enriched diet for 15 weeks, followed by characterization of intestinal phenotype. We observed an increase in the number of resident and/or recruited macrophages in the *lamina propria* of the small intestine of treated mice (Additional file 3: Figure 4 A). We then evaluated goblet cell number and found a significant reduction in treated mice (Fig. 4A), evidencing also in this model hallmarks of intestinal inflammation following BSSG dietary uptake. Ultrastructural analysis revealed that BSSG-fed mice exhibited significantly shorter microvilli compared to controls (Fig. 4B), potentially implying impaired enteric absorption capacity. Consistently, treated mice weighed less than controls (Fig. 4C) despite similar food intake (Additional file 3: Figure 4B).

RNAseq analysis of gut tissue revealed 1835 differentially expressed genes between treated and untreated mice (982 upregulated and 853 downregulated in treated animals). Upregulated genes were primarily associated with *regulation of immune system process* and *immune response* (Fig. 4D, E; Additional file 3: Figure 4 C, left panel) and were related with *actin cytoskeleton*, *side of membrane*, and *microvillus* (Additional file 3: Figure 4D), thus confirming that BSSG directly impacts on genes involved in enterocytes’ brush border structure. Conversely, downregulated genes were involved in *small molecules metabolic process, regulation of cell growth*, *heme*/*porphyrin-containing compound biosynthesis,* and *regulation of neuron projection development* (Fig. 4Dʹ, E), and were associated with *glutathione transferase activity* and *transmembrane transporter activity* (Additional file 3: Figure 4 C, right panel). RT-qPCR analysis confirmed the activation of inflammatory and immune responses, evidencing upregulation of *Nod1* (*NOD-like receptor 1*), *Tlr-2, Tlr-4, Tlr-6* (*Toll-like receptor-2, −4, −6*), *Nlrp3* (*NLR family pyrin domain containing 3*), *Il-1β* (*Interleukin-1β*) and *Ifn-γ* (*Interferon-γ*) and downregulation of *Ikb-α* (*NF-kappa-B inhibitor alpha*) and *Reg3-γ* (*Regenerating islet-derived protein 3-γ*)*.* We also observed upregulation of *Plp1* (*Proteolipid protein 1*), which is highly expressed in enteric glia (Fig. 4F).

Finally, analysis of fecal microbiota revealed some differences in bacterial composition between BSSG-fed mice and controls. Treated mice showed a higher relative abundance of potentially pathogenic bacterial families, including *Bacteroidaceae, Helicobacteraceae* and *Prevotellaceae*, typically associated with intestinal inflammation, and reduced anti-inflammatory taxa such as *Lachnospiraceae* (Additional file 3: Figure 4E). As observed in zebrafish, these data may reflect early alterations in gut microbiota in this pre-symptomatic ALS-PDC mouse model. However, no definitive conclusions can be drawn due to the lack of statistical significance, likely resulting from the limited sample size.

### BSSG interaction with the glucocorticoid receptor as a possible mechanism of action

To investigate a possible mechanism of action of BSSG, we examined whether it could interact with known steroid hormones receptors, given its structural similarity to these molecules. A radioligand binding assay demonstrated that BSSG determined a 12.5% inhibition of the binding between glucocorticoid receptor (GR) and its radiolabelled specific ligand ([^3^H]-dexamethasone) and a 4.7% inhibition for the androgen receptor (AR) and its radiolabelled specific ligand ([^3^H]-methyltrienolone). No interference with estrogen receptor (ER), mineralcorticoid receptor (MR) or progesterone receptor (PR) was observed (Additional file 3: Figure 3 C).

**Fig. 5:**
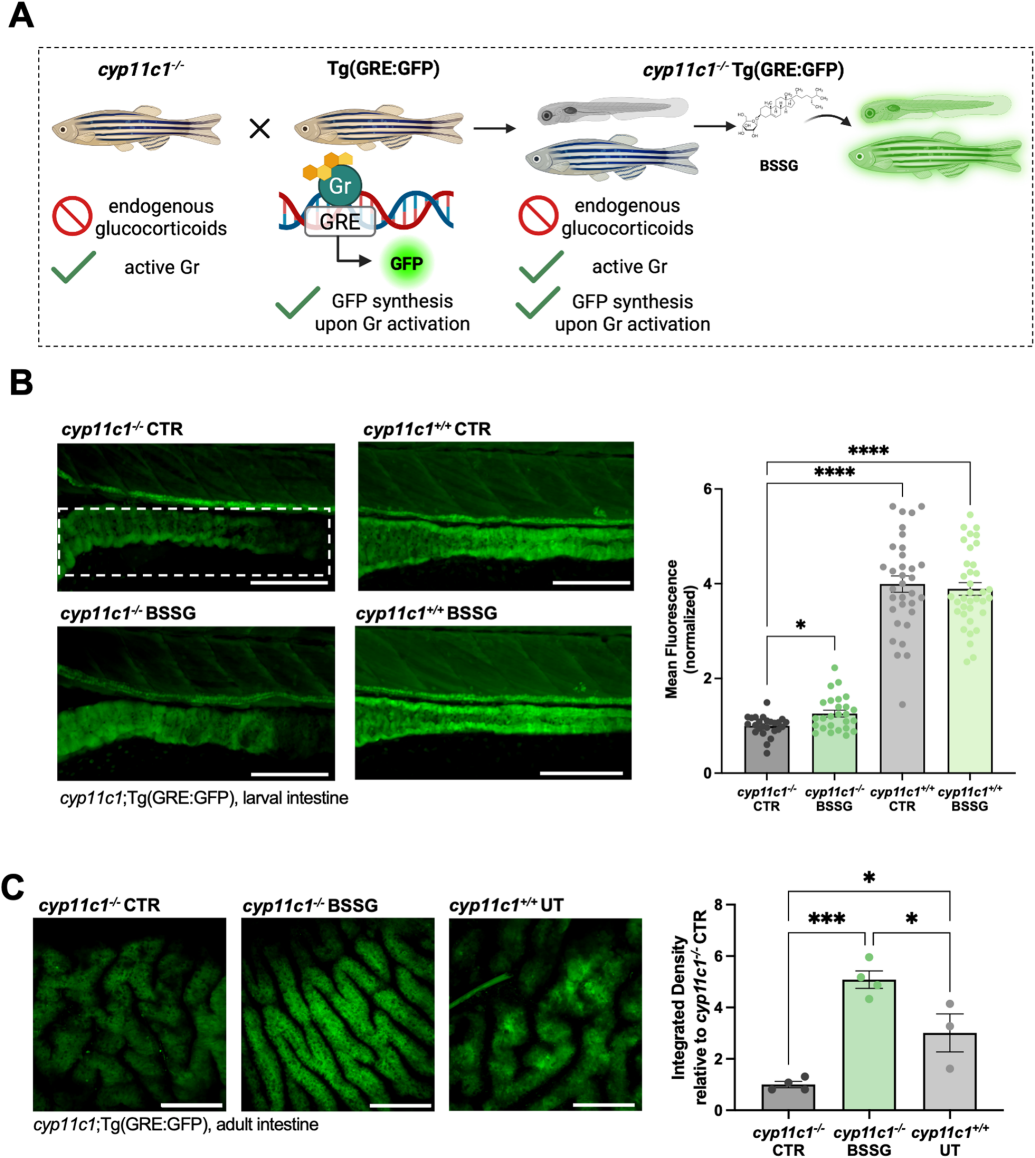
In vivo BSSG interaction with the glucocorticoid receptor. **A** Schematic representation of the mutant and transgenic zebrafish lines crossed to obtain *cyp11c1*^*−/−*^;Tg(GRE:EGFP) individuals. **B** Representative magnification of the mid-intestine of *cyp11c*^+/+^ and *cyp11c1*^*−/−*^*;*Tg(GRE:EGFP) zebrafish larvae and mean fluorescence quantification in treated larvae compared to controls. Dotted lines evidence the analysed region. N = 3 biological replicates. **C** Representative 20× confocal acquisitions of GC-responsive intestine in adult UT (untreated) *cyp11c1*^+/+^ and *cyp11c1*^*−/−*^*;*Tg(GRE:EGFP) after 15-days feeding with BSSG-enriched food and Integrated Density quantification. N ≥ 3 animals/condition. Bar graphs show the mean ± SEM. Statistical analysis was performed using one-way ANOVA. *P < 0.05; ***P < 0.001; ****P < 0.0001. Scale bar: 200 µm

To verify if BSSG effectively binds Gr in vivo, we exploited the *cyp11c1* zebrafish mutant line recently generated in our laboratory. As other published *cyp11c1* (*cytochrome P450 family 1 subfamily C member 1*) zebrafish mutant lines, homozygous *cyp11c1*^*−/−*^ cannot synthetize active glucocorticoids (GCs), while retaining a functional Gr [40, 41]. To visualize Gr activity, this *cyp11c1* mutant line was crossed with transgenic Tg(GRE:EGFP) line, which expresses GFP after Gr activation [21] (Fig. 5A). Thus, *cyp11c1*^*−/−*^;Tg(GRE:EGFP) animals, lacking endogenous GCs, allow to visually discriminate the effective binding of BSSG with the Gr in vivo. Following BSSG treatment, *cyp11c1*^*−/−*^;Tg(GRE:GFP) larvae showed a significant increase in intestinal fluorescence, suggesting that BSSG can effectively modulate Gr activation (Fig. 5B). The same analysis performed in adult transgenic mutants exposed to BSSG-enriched diet also revealed a significant increase of intestinal fluorescence (Fig. 5C). The quantification of gut fluorescence in *cyp11c1*^+/+^; Tg(GRE:GFP) larvae and adults showed, as expected, a higher baseline signal compared with *cyp11c1*^*−/−*^;Tg(GRE:GFP), due to constitutive activation of Gr by endogenous glucocorticoids [21]. Accordingly, BSSG treatment in *cyp11c1*^+/+^;Tg(GRE:GFP) larvae failed to induce any further detectable increase of intestinal fluorescence.

### Gr deficiency reduces the negative effects of BSSG on gene expression

**Fig. 6:**
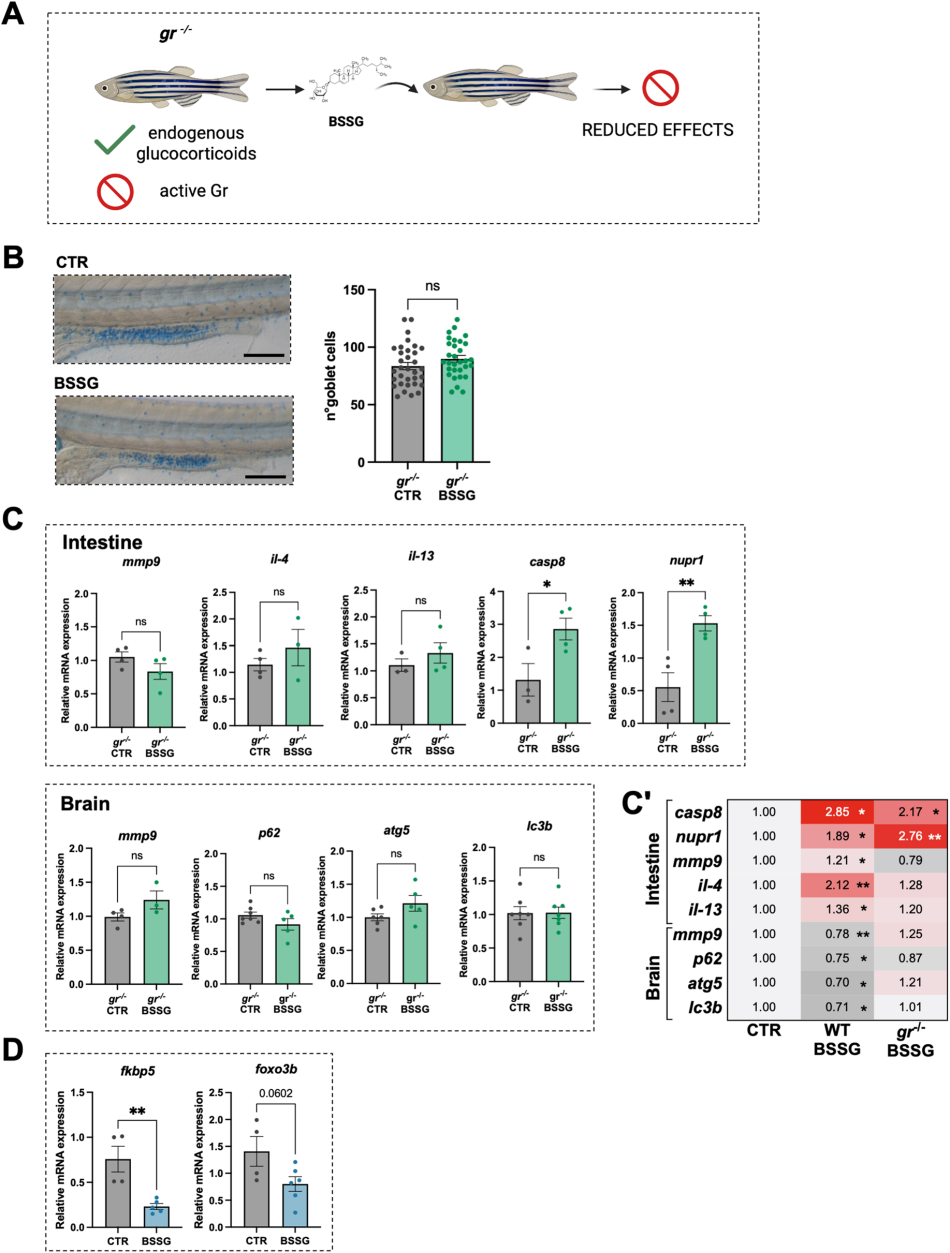
Goblet cell analysis and gene expression in *gr*^*−/−*^ zebrafish line. **A** Schematic representation of the experimental setup exploiting *gr*^*−/−*^ adult zebrafish. **B** Analysis of goblet cells number stained with alcian blue in the mid-intestine of *gr*^*−/− *^CTR and BSSG-treated larvae. **C** RT-qPCR analysis of different inflammation-, stress- and autophagy-related genes in RNA samples from adult *gr*^*−/−*^zebrafish intestines (upper panel) and brains (lower panel). **Cʹ** Schematic summary of the differential expression of key markers in the intestine and brain of WT and *gr*^*−/−*^ adult zebrafish. Cell numbers indicate the amount of mRNA and the statistical significance (asterisks) relative to the respective untreated WT or *gr*^*−/−*^, which were both set to 1. **D** RT-qPCR analysis of Gr-modulated genes in the intestine of WT adult zebrafish. Each dot represents a tissue sample from a single individual. N ≥ 3 animals/condition. Bar graphs show the mean ± SEM. Statistical analysis was performed using unpaired Student’s t-test. *P < 0.05; **P < 0.01; *ns*, not significant

To confirm the role of the Gr in mediating the action of BSSG, we exploited the *nr3c1*^*ia30/ia30*^ zebrafish mutant line (hereafter called *gr*^*−/−*^), previously generated in our laboratory, in which the *gr* gene has been knocked out [17] (Fig. 6A).

Interestingly, no difference in the number of goblet cells was observed in BSSG-treated *gr*^*−/−*^ larvae compared to controls (Fig. 6B), in contrast to what observed in treated WT individuals (Fig. 1C). Similarly, no significant alterations were detected in the expression of *mmp9, il-4* and *il-13* in the intestine of adult *gr*^*−/−*^ zebrafish fed with BSSG-enriched diet. These findings suggest that BSSG likely interferes with the anti-inflammatory Gr activity. Conversely, the expression of *casp8* and *nupr1* was significantly upregulated, similarly to what was observed in WT animals, indicating that BSSG likely interferes with multiple pathways and cellular targets beyond Gr (Fig. 6C upper panel and Fig. 6C’).

Interestingly, also mRNA levels of *mmp9, p62, atg5* and *lc3b* did not differ between brains of treated and control *gr*^*−/−*^ zebrafish (Fig. 6C lower panel and Fig. 6Cʹ).

Following in vivo evidence of a potential interaction between BSSG and Gr, we investigated whether BSSG could also impact on the fine-tuned regulation of gene expression commonly mediated by GCs/Gr signalling. We therefore evaluated the expression of Gr-target genes such as *fkbp5* (*FKBP prolyl isomerase 5*) and *foxo3b* (*forkhead box protein O 3b*) in the intestine of WT adults fed with BSSG. We observed a significant reduction in *fkbp5* expression, while *foxo3b* displayed a decreasing trend in treated individuals (Fig. 6D), further supporting our hypothesis.

### Gr deficiency mitigates the alteration of intestinal muscular contractility and microbiota composition

**Fig. 7:**
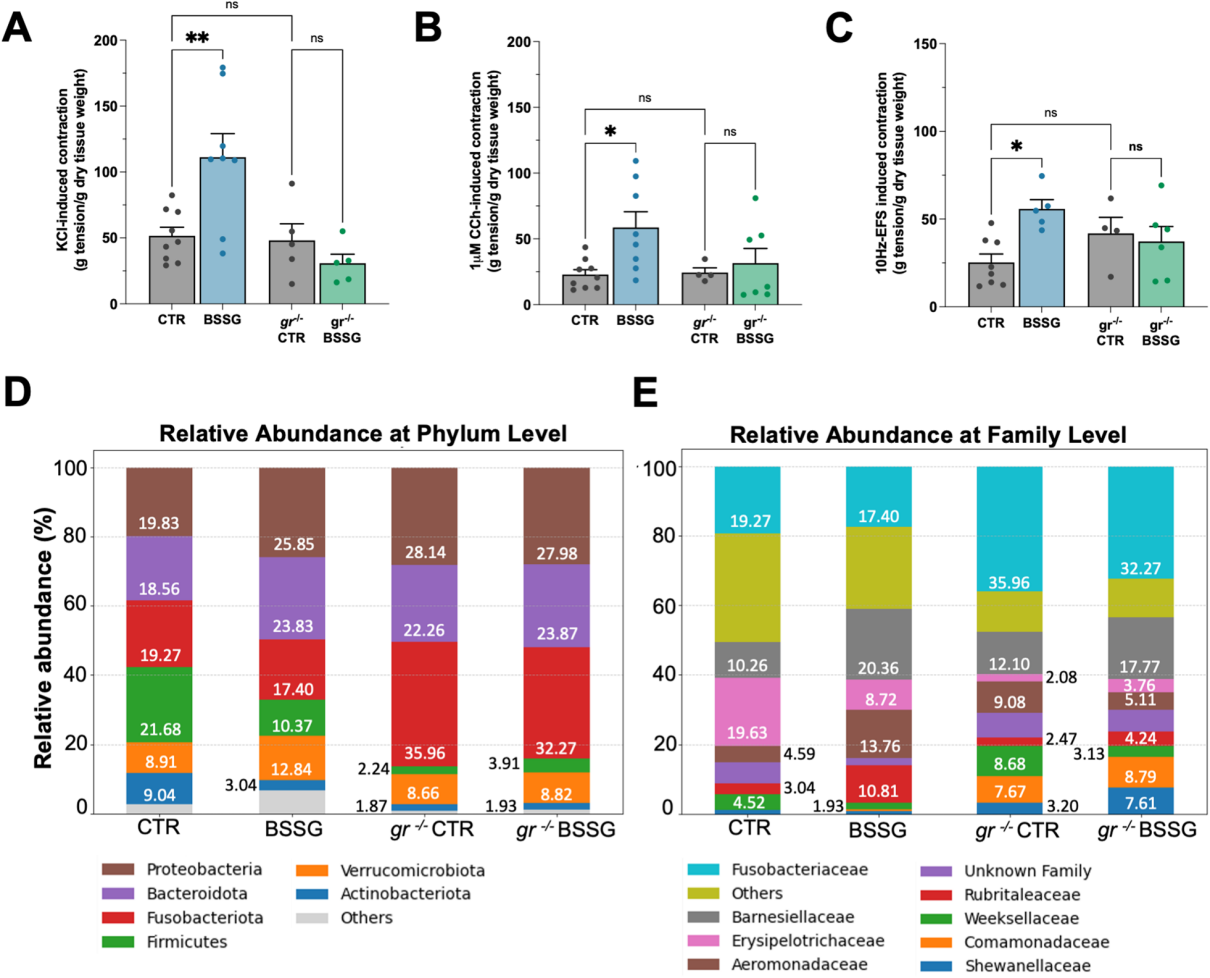
Analysis of intestinal motility and gut microbiota in *gr*^*−/−*^ zebrafish line. **A–C** Ex vivo analysis of intestinal contractile responses elicited by 40 mM KCl (**A**); 1 µM carbachol (CCh) (**B**); 10 Hz EFS (**C**), in isolated intestinal preparations of WT (left side of the graphs) and *gr*^*−/−*^ (right side of the graphs) adult zebrafish with or without BSSG treatment. Data are reported as mean ± SEM. Each dot represents a tissue sample from a single individual. N ≥ 4 animals/condition. Statistical analysis was performed using one-way ANOVA. *P < 0.05; **P < 0.01; ns, not significant. **D, E** Analysis of adult zebrafish gut microbiota. Bar graphs show the percentage of the relative abundance in bacterial phyla (**D**) and families (**E**) in both WT (left side of the graphs) and *gr*^*−/−*^ (right side of the graphs)

To evaluate whether the impact of BSSG on intestinal neuromuscular function is modulated by Gr, we applied the ex vivo approach in *gr*^*−/−*^ adult zebrafish after administration of BSSG-enriched diet. We observed that gut samples from *gr*^*−/−*^ animals did not show any variation in muscle- and neuron-induced contractility when exposed to KCl, 1 µM CCh and 10 Hz EFS after BSSG treatment, maintaining responses comparable to untreated *gr*^*−/−*^ controls (Fig. 7A–C; for concentration–response curves see Additional file 3: Figure 2D, E). These data point out that defects in gut motility could be influenced by BSSG interaction with Gr and are abolished in *gr*^*−/−*^ mutant line.

Moreover, BSSG treatment appeared to induce fewer changes in the gut microbiota composition of *gr*^−/−^ animals compared with those reported for WT (Fig. 7D, E). At the phylum level, no evident alterations were detected, except for a slight increase in *Firmicutes* (Fig. 7D, right side). At the family level, we observed similar abundance of *Barnesiellaceae*, *Erysipelotrichaceae* and *Rubritaleaceae,* suggesting that BSSG exposure has a limited impact on these bacterial strains in *gr*^−/−^ individuals (Fig. 7E, right side).

It should be noted that the lack of the Gr is associated with baseline differences in gut microbiota composition. In particular, *Fusobacteriota* were more abundant in *gr*^*−/−*^ animals, whereas *Firmicutes* and *Actinobacteriota* were less represented, in opposition to what observed in WT. At the family level, *Fusobacteriaceae, Comamonadaceae* and *Shewanellaceae* were more abundant in *gr*^*−/−*^ than in WT. Nevertheless, our findings suggest that BSSG influences the gut microbial community differently in *gr*^*−/−*^ animals compared to WT, as also supported by 2D PCoA analysis and ASVs (Amplicon Sequence Variants) relative abundance calculated across all experimental groups (Additional file 3: Figure 5), where *gr*^*−/−*^ individuals appear more similar to each other regardless of BSSG treatment.

## Discussion

In this work, we used the zebrafish and mouse models to characterize the link between BSSG intake and the onset of the complex neurodegenerative disorder known as ALS-PDC.

We found that BSSG is effectively absorbed by zebrafish and mice, where it triggers a wide range of effects related to intestinal dyshomeostasis. A damaged gut epithelial barrier, a pro-inflammatory profile, impaired gut functionality and microbiota alteration are all hallmark features of a perturbed gut-brain axis, that connects the ENS and the CNS [42]. Therefore, we hypothesize that BSSG may affect this route, by inducing gut inflammation that could predispose to neurodegeneration.

The pro-inflammatory activity seemed to be specifically caused by BSSG, in agreement with the reported toxicity associated with the presence of a single glucidic group [43], since β-sitosterol did not elicited inflammatory effects on LREs, a marker of intestinal inflammation in different zebrafish models [44, 45]. Gut dyshomeostasis was further supported by a reduced number of goblet cells and decreased expression of *agr2*, coding for a protein essential for intestinal mucus production [46]. Similar alterations are known to promote intestinal inflammation by weakening the epithelial barrier [47]. Furthermore, neutrophils recruitment to the mid-intestine, activation of the NF-κB pathway, increased apoptosis, reduced proliferative capacity of intestinal stem-like cells, and upregulation of immune-related genes collectively indicate an ongoing inflammatory response (Fig. 1). Indeed, *mmp9* and *stat3* play a role in immune activation [48], while enteric *pept1* is stimulated by pro-inflammatory cytokines. Noteworthy, the expression of these genes is increased in inflammatory bowel disease (IBD) patients and related animal models [49–52].

Chronic administration of BSSG to zebrafish larvae and adults confirmed the intestinal inflammatory phenotype, as emerged from transcriptomic analysis (Fig. 3). In addition to *mmp9* and *pept1*, we highlighted the upregulation of *saa*, involved in the activation of NF-kB signalling, in the promotion of downstream genes such as *mmp9* and in the regulation of neutrophils migration [53]. Similarly, *s100a10a,* expressed in the digestive tract, drives neutrophil recruitment in a larval model of intestinal infection [54]. The S100a10a protein is considered homolog of human Calprotectin, another marker for IBD [55]. Interestingly, it is also increased in stool samples from PD and Alzheimer’s disease (AD) patients [56].

Based on these results and according to previous studies [11], we developed an ALS-PDC pre-symptomatic mouse model, focusing on the small intestine of BSSG-treated animals (Fig. 4). As observed in zebrafish, RNAseq analysis of mouse small intestine revealed that most of the DEGs were involved in the modulation of immune response. Toll-like receptors (TLRs) were significantly upregulated: *Tlr-2*, *Tlr-4* and *Tlr-6*, along with NOD-like receptors, have been associated with the activation of NF-kB pathway [57] and IL-1β production [58]. Moreover, the observed alteration of several players involved in NF-kB pathway, Nlrp3 assembly and inflammasome activation [59], together with the downregulation of *Ikb-α*, which normally provides negative-feedback to modulate inflammatory response, suggested an impairment of this pathway after BSSG treatment, leading to chronic inflammation. Consistently, patients and animal models with IBD show increased expression of TLRs, persistent NF-kB and NLRP3 activation and elevated levels of pro-inflammatory cytokines such as IL-1β and IFN-γ [57]. Interestingly, increased expression of these same pro-inflammatory genes has been observed in intestinal biopsies of PD patients [60], further supporting a link between intestinal inflammation and neurodegenerative disease predisposition. Activation of the immune response following BSSG dietary intake was further supported by the increased presence of macrophages in the *lamina propria* of treated mice (Additional file 3: Figure 4 A).

Moreover, the antimicrobial peptide REG3-γ, involved in proper distribution of the mucus layer [61], was downregulated in our model. Coherently, we observed a reduction in the number of mucus-producing goblet cells, as already observed in treated zebrafish larvae. Among the upregulated mouse DEGs, we found *Lrrk2* (*Leucine-rich repeat kinase 2*), one of the most relevant PD genetic risk factors. In the intestine, LRRK2 protein is involved in the immune system activation through positive regulation of NF-kB, and its increased expression has been associated with pro-inflammatory effects in IBD mouse models as well as in PD patients, as reviewed in [62].

Our zebrafish model provided evidence that this inflammatory condition is also associated with marked alterations in gut physiology, observed after both acute and chronic exposure to BSSG (Fig. 2A–F). Dysregulation of peristalsis and delayed gastrointestinal transit are well-recognized hallmarks of IBD [63] and have also emerged as prodromal symptoms in autistic spectrum disorders [64, 65] and neurodegenerative diseases such as PD and ALS [66, 67]. Indeed, constipation can affect patients many years before the appearance of the typical motor symptoms. The innovative ex vivo analysis of gut contractility, applied for the first time to adult zebrafish intestines, provided strong evidence that ENS functionality is affected by BSSG consumption. Several findings link intestinal muscular hypertrophy and hypercontractility with infection and increased expression of pro-inflammatory cytokines. Among them, IL-4 and IL-13 are responsible for higher intestinal smooth muscle contraction in mice during gut inflammation or enteric infection [68, 69]. Moreover, a mouse model of DSS (dextran sulphate sodium)-induced colitis showed increased neuromuscular contraction upon CCh stimulation [70]. Collectively, these data demonstrate that BSSG administration affects both muscular and neuronal components of the gut, altering gastrointestinal motility and potentially damaging enteric neurons.

Preliminary analyses of gut microbiota in treated adult zebrafish (Fig. 2G, H) revealed a higher abundance of *Proteobacteria* and a reduction in *Firmicutes*, in agreement with findings from TNBS (trinitrobenzene sulfonic acid)-induced intestinal inflammation [71] and IBD patients [72, 73]. Furthermore, a reduced *Firmicutes*/*Bacteroidetes* ratio has been already associated with IBD [36], while alterations in the relative abundance of bacterial phyla/families similar to the ones presented in this work were described for zebrafish exposed to contaminants leading to dysbiosis [74]. Noteworthy, comparable gut microbiota alterations have also been observed in neurodegenerative diseases such as AD, ALS and PD, where changes in bacterial communities may precede disease onset [75].

In line with the gut–brain axis hypothesis, we investigated if BSSG exerted detrimental effects on the CNS in our zebrafish model. In the brains of treated adult zebrafish, we found decreased expression of autophagy-related genes, suggesting possible impairment of the autophagic process. This dysfunction alone can induce neurodegeneration by promoting the accumulation of neurotoxic proteinaceous aggregates [76]. However, this aspect requires more extensive and tailored analyses.

Data obtained using zebrafish mutant lines lacking genes involved in GCs synthesis (*cyp11c1*) and signalling (*gr*), suggested that BSSG may exert part of its effects by interacting with the Gr. In particular, increased fluorescence in treated *cyp11c1*^*−/−*^;Tg(GRE:GFP) zebrafish intestine (Fig. 5) suggests that this sterol-derived molecule can interfere with Gr nuclear translocation and activity. Moreover, the absence of significant changes in goblet cells number in *gr*^*−/−*^ treated larvae, the presence of only minor gene expression alterations in intestines and brains of *gr*^*−/−*^ adult zebrafish fed with BSSG, and their unchanged intestinal neuromuscular activity observed ex vivo (Figs. 6 and 7), indicate that inflammatory and neuromuscular signalling could be, at least partially, mediated by BSSG interaction with Gr. Both *fkbp5* and *foxo3b* are directly regulated by Gr, modulating sensitivity to GCs and resolving inflammation through the inhibition of NF-kB pathway [77], respectively. Their reduced expression in the intestine of treated WT zebrafish (Fig. 6D), may result from local impairment of Gr function, supporting the hypothesis of reduced anti-inflammatory activity after BSSG exposure. Notably, recent evidence show that the absence of intestinal GR in DSS-treated mice exacerbated inflammatory response, highlighting the protective role of GR activity against IBD [78]. However, despite our extensive in vivo findings, this compelling hypothesis requires further in vitro validation due to the complexity of steroid receptor signaling. Nevertheless, altered GCs levels and impaired GR regulation have already been linked with pathogenesis and progression of neurodegenerative diseases like ALS, PD and AD [79], further supporting the proposed mechanism through which BSSG, by targeting GR, may contribute to ALS-PDC etiology.

## Conclusions

To conclude, this work reveals that increased levels of dietary BSSG induce a marked intestinal inflammation that has never been previously described. Our results suggest that this molecule initially affects the enteric district and lately the CNS, promoting neurodegeneration that may culminate in ALS-PDC occurrence. This interplay between the intestine and the CNS underscores the relevance of the gut–brain axis. Furthermore, BSSG interaction with the GR suggests a possible modulation of its anti-inflammatory activity, highlighting the importance of physiologic function of GCs in the intestine, where they regulate immune homeostasis. According to the gut–brain axis paradigm, interference with such functions may exacerbate inflammation, possibly leading to neurodegeneration. Therefore, restoring intestinal homeostasis could represent a potential early intervention strategy to prevent disease progression.

## Supplementary Information

Additional file 1. BSSG synthesis. Detailed description of BSSG chemical synthesis, quality control and NMR analysis

Additional file 2. Primers tables. Primers.

Additional file 3. Figure S1. A) Fish embryo acute toxicologyTest on zebrafish larvae treated with increasing concentrations of BSSG and of the vehicle DMSO. B) Measure of larval morphological traits: standard length, eye area and area of the swimming bladder. N =3 biological replicates, each consisting of at least 10 larvae. Bar graphs show the mean ± SEM. Statistical analysis was performed using unpaired Student’s t-test. *ns*, not significant. C) Mass spectrometry analysis of lipid extracts from pooled heads and trunks of 5 dpf treated and control larvae. Numbers above the bars indicate the achieved internal concentration of BSSG. Figure S2. A) Magnification of the zebrafish mid-intestine region stained in vivo with neutral red and quantification of its length in larvae treated with 10 µM β-sitosterolcompared to controls. N = 4 biological replicates, each consisting of at least 10 larvae. Scale bar: 200 µm. B) RT-qPCR analysis of autophagy-related genes in pooled 5-dpf CTR and BSSG-treated larvae. N ≥3 biological replicates. Data are expressed as mean ± SEM. Statistical analysis was performed using unpaired Student’s t-test: *P<0.05; ***P<0.001. C) Immunofluorescence staining of HuC/D+ enteric neurons and Sox10+ neuronal progenitors in 5-dpf zebrafish larval intestine. Bar graphs show the mean ± SEM. Statistical analysis was performed using unpaired Student’s t-test. ns, not significant. Scale bar: 200 µm. D-E) *Ex vivo* concentration-response curves to carbachol stimulationand electric field stimulationin isolated ileal preparations of WTand mutant gr^-/-^ adult zebrafishwith or without BSSG *in vivo *treatment. N ≥4 animals/condition. Statistical significance was calculated with a two-way ANOVA followed by a Bonferroni post hoc test for multiple comparisons. *P<*0.05*; ***P<0.001. Figure S3. A) Bar charts with RNAseq analysis from RNA samples of pooled 30 dpf chronically treated whole zebrafish larvae. GO Molecular function enrichment for the up- and downregulated genes. B) GO Cellular component enrichment for downregulated genes. C) Schematic representation of the radioligand binding assay performed for each steroid hormone receptor with its specific radiolabelled ligand. The heatmap indicates the percentage of inhibition of GR binding to its radiolabelled specific ligand following the interaction with BSSG. Figure S4. A) Immunofluorescence staining with the macrophage marker F4/80 on mouse small intestine histological sections. Arrowheads indicate macrophages in the *lamina propria*. Dotted lines define the outlines of the samples. Bar graph shows the mean ± SEM. Statistical analysis was performed on 5 sections obtained from 3 animals/condition using unpaired Student’s t-test. *P<0.05. Scale bar: 200 µm. B) Representative plot of the cumulative amount of foodconsumed by BBSG-treated and control mice during the whole experimentation period. Food leftovers were weighed weekly to keep track of the amount of BSSG ingested by the animals through the feed. C)Bar charts with RNAseq analysis of RNA samples extracted from mouse small intestine. GO Molecular function enrichment of up- and downregulated genes. D) GO Cellular component enrichment of up- and downregulated genes. E) Analysis of mice fecal microbiota. The bar graph shows the percentage of relative abundance of bacterial families in N =2 animals/condition. Figure S5. A) Principal Component analysisof adult zebrafish gut microbiota analysed in four different conditions: WT control, WT BSSG, *gr*^*-/-*^ CTR and, *gr*^*-/-*^ BSSG. The firstand the second principal componentsare shown on the horizontal and vertical axes, respectively. B) Average ASVs in the four conditions. The number of ASVs and the Shannon index are plotted. C) Heatmap plotting the relative abundance of the ASVs that reach at least the 0.5%.

Additional file 4. List of zebrafish DEGs. Table with the complete list of zebrafish differentially expressed genes

Additional file 5. List of mouse DEGs. Table with the complete list of mouse small intestine differentially expressed genes

## Data Availability

Transcriptional data generated during the current study have been deposited in SRA database (URL will be made available upon request). Other data supporting the findings of this study are available on reasonable request to the corresponding authors.

## Abbreviations

AD: Alzheimer’s disease
ALS: Amyotrophic Lateral Sclerosis
ALS-PDC: Amyotrophic Lateral Sclerosis-Parkinsonism Dementia complex
AR: Androgen receptor
BSSG: β-Sitosterol β-d-glucoside
CCh: Carbachol
cyp11c1: Cytochrome P450 Family 1 Subfamily C Member 1
CNS: Central nervous system
DMSO: Dimethyl sulfoxide
DSS: Dextran sulphate sodium
EFS: Electrical field stimulation
ENS: Enteric nervous system
ER: Estrogen receptor
GCs: Glucocorticoids
Gr: Glucocorticoid receptor
IBD: Inflammatory bowel disease
KCl: Potassium chloride
LRE: Lysosome-rich enterocyte
MR: Mineralcorticoid receptor
PCoA: Principal Component Analysis
PD: Parkinson’s disease
PR: Progesterone receptor
TNBS: Trinitrobenzene sulfonic acid
WT: Wild type
α-GlcChol: Glucosyl-α-d-cholesterol
β-GlcChol: Glucosyl-β-d-cholesterol
