# Supplementary material for: β-Sitosterol β-d-glucoside (BSSG) triggers intestinal inflammation in zebrafish and mouse models prior to neurodegeneration onset
Source: J Biomed Sci. 2026 May 4;33:45. doi: 10.1186/s12929-026-01249-8 (PMC13137512; doi:10.1186/s12929-026-01249-8)
Supplement: Supplementary file 1 — Additional file 1. BSSG synthesis. Detailed description of BSSG chemical synthesis, quality control and NMR analysis [file 12929_2026_1249_MOESM1_ESM.docx]

**β-SITOSTEROL β-D-GLUCOSIDE SYNTHESIS**

β-sitosterol β-D-glucoside (***β*-1)** was synthesized starting from the commercially available sitosterol (75% purity, 85451- Sigma) that contained minor impurities due to other phytosterols like campesterol and stigmasterol (**Scheme** **1**).


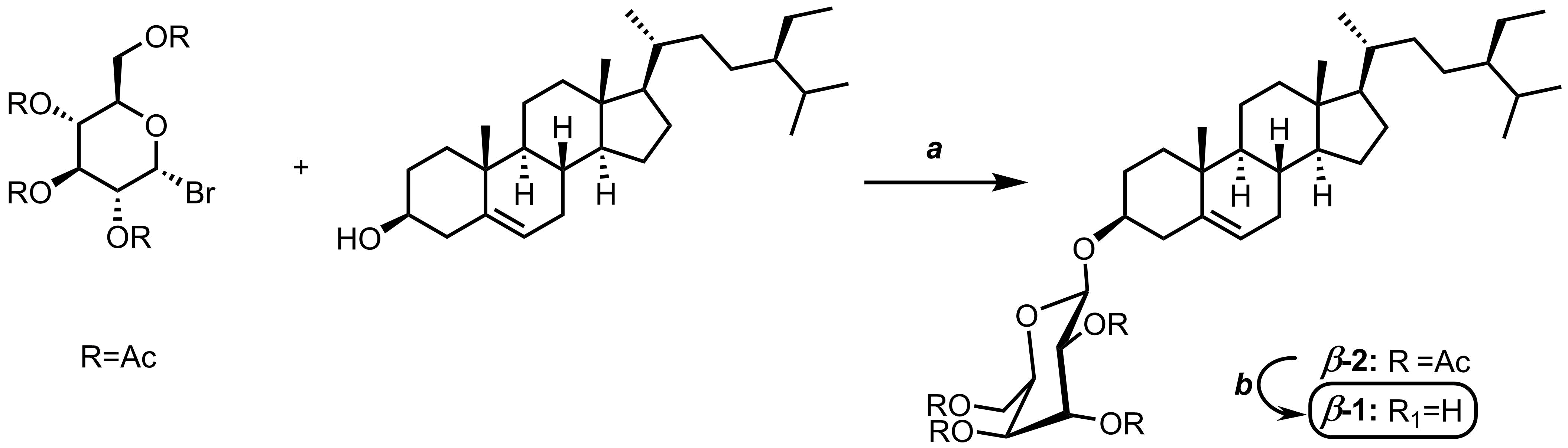


**Scheme 1.** Synthesis of ***β*-1**. Reagents and conditions: (a) Ag_2_O, MgSO_4_ anhydrous Et_2_O, inert Ar atmosphere, 16 h, r.t., 31%; (b) MeONa-MeOH, 3 h, 63%.

The first synthetic step involved the glycosidic coupling of sitosterol starting from activated 1-bromo-*α-D*-glucose tetraacetate,^^[[1]](#endnote-1)^^ mediated by Ag_2_O leading to the product ***β*-2** with 31% yield after chromatographic purification (for details, see Supporting Information).

The target product ***β*-1** was obtained after acetyl deprotection of ***β*-2** using a catalytic amount of MeONa in MeOH,^^[[2]](#endnote-2)^^ with 63% yield after trituration of the crude compound with EtOAc. High Resolution ESI-MS analysis confirmed the structure of ***β*-1** observing a m/z value of 599.4247 a.m.u. corresponding to the [M+Na^+^] ion. ^1^H and ^13^C{^1^H} NMR spectra were in agreement with those reported in the literature in the same deuterated solvent dmso-*d_6_*.^^[[3]](#endnote-3)^^ The chemical shift value and coupling constant of the anomeric H atom (4.21 ppm, *J*= 8 Hz) confirmed the *β* configuration of ***β*-1**.^3^ Moreover, no resonance signals corresponding to the *α*-anomer of the glucosyl sitosterol were detected. The purity of ***β*-1** (98.1%) was determined by ^1^H NMR integration with 1,4-dimethoxybenzene as internal reference using the qNMR Mestrenova® plugin software.^^[[4]](#endnote-4)^^

### **General procedures and materials**

Reagents and solvents with high purity degree purchased by the providers were used as received. Otherwise, they were purified following the procedures reported in the literature.^[[5]](#endnote-5)^ Anhydrous solvents were prepared by adding activated 4 Å molecular sieves to the solvent under inert atmosphere. All the reactions were carried under Ar atmosphere. Molecular sieves were activated shortly before the use by continuous heating at 150 °C under *vacuum*. The reactions were followed with TLC Polygram® Sil G/UV_254_, 0.25 mm thickness. Flash chromatography was performed with silica gel Merk 60 (230-400 mesh) following procedures reported in literature.^[[6]](#endnote-6)^

^1^H NMR, ^13^C{^1^H} NMR, and 2D spectra were recorded with Ascend 400 spectrometer, operating at 400 and 100 MHz respectively. Resonance frequencies are referred to tetramethylsilane, chemical shifts are expressed in ppm with reference to the deuterated solvent residual peak. Multiplicity is addressed as s (singlet), d (doublet), dd (doublet of doublets), t (triplet), m (multiplet), and so on. The NMR and IR Spectra were elaborated using Mestrenova® software.

High resolution mass spectrometry measurements were performed using a Bruker Compact Q-TOF. The compounds studied were dissolved in methanol with a concentration of 1 ppm. The product **β-1** was injected into the ESI source by direct infusion with a syringe pump integrated in the mass spectrometer. Mass spectra were acquired in positive-polarity mode. The instrumental conditions were as follows: Nebulizer 0.4 Bar, Capillary 4500 V, Dry Heater 180 °C, Scan Begin 50 m/z, End Plate Offset -500 V, Dry Gas 4.0 l/min, End 1300 m/z, Collision Cell RF 650.0 Vpp. The HRMass mass spectra were elaborated with DataAnalysis® software.

### **Quantitative NMR**

Quantitative NMR was performed by dissolving 9.81 mg of analyte **β-1** and 3.55 mg of internal reference 1,4-dimethoxybenzene in 1 ml of DMSO-d_6_. The analysis was done with three replicates. Instrumental parameters was: *Delay[s]* 5.0894; *Number Of Scans* 64; *Acquisition size* 32768; *Processed number of points* 65536; *Apodization***:** Stanning Apodization: 8.00; *Phasing* Method: Manual; InitMode: InitIncremental; Ph0: -3.30; Ph1: 0.29; *Baseline Correction* Algorithm: Bernstein; PolyOrder: 5.00; *Sample Integration* Method: Sum; Growth Factor: 25.00; *Reference Integration* Method: Sum; *Peak Picking* Method: qGSD.

**Experimental procedures - Syntheses**

*(2R,3S,4R,5R,6R)-2-(acetoxymethyl)-6-(((3S,8S,9S,10R,13R,14S,17R)-17-((2R,5R)-5-ethyl-6-methylheptan-2-yl)-10,13-dimethyl-2,3,4,7,8,9,10,11,12,13,14,15,16,17-tetradecahydro-1H-cyclopenta[a]phenanthren-3-yl)oxy)tetrahydro-2H-pyran-3,4,5-triyl triacetate*


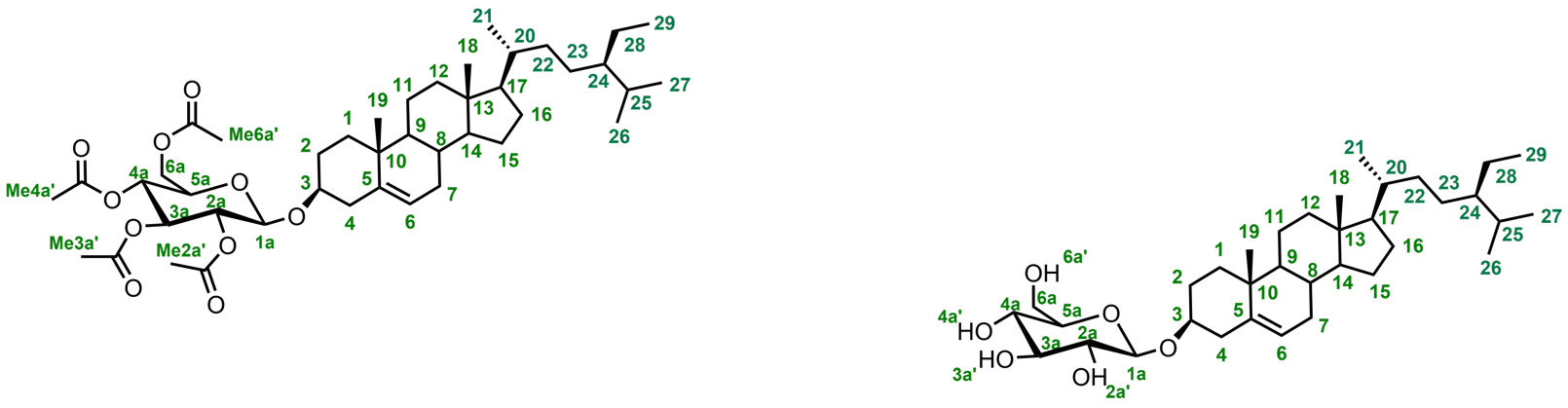


**β-2**

In a two necked flask, *b*-sitosterol (5 g, 12 mmol), freshly precipitated Ag_2_O (7.56 g, 33 mmol), 4 Å molecular sieves, and MgSO_4_ dried under vacuum at 250 °C (10 g, 84 mmol) were added. The mixture was treated with several vacuum and Argon cycles. Subsequently dry Et_2_O (36 mL) was added. 1-bromo-*α-D*-glucose tetraacetate dissolved in Et_2_O (36 mL) was added dropwise in 5 min. The mixture was stirred for 5 h, then another portion of Ag_2_O (7.56 g, 33 mmol) was added and the resulting mixture was stirred for an additional two days at room temperature. In order to remove inorganic salts, the mixture was filtered on a pad of Celite, washing thoroughly with Et_2_O and the ethereal phases were collected and dried under vacuum. The crude product obtained by solvent removal corresponding to 8.2 g was purified by flash chromatography (Eluent 8:2 Cy/EtOAc) leading to 3.35 g of purified product **β-2** corresponding to 31% yield. The ^1^H NMR chemical shifts in chloroform-*d* were consistent with those published in the literature.^3^

^1^H NMR (400 MHz, CDCl_3_) δ 5.38 (m, 1H, H_6_), 5.22 (t, *J* = 2.3 Hz, 1H, H_3a_), 5.10 (t, 1H, H_4a_), 4.98 (dd, *J* = 3.0, 8.0 Hz, 1H, H_2a_), 4.61 (d, *J* = 1.6 Hz, 1H, H_1a_), 4.28 (dd, *J* = 4.8, 12.2 Hz, 1H, H_6a''_), 4.15 (m, 1H, H_6a'_), 3.70 (m, 1H, H_5a_), 3.51 (m, 1H, H_3_), 2.27 (m, 2H, H_4_), 2.10 (s, 3H, Me_6a'_), 2.07 (d, *J* = 0.5 Hz, 3H, Me_4a'_), 2.04 (m, 3H, Me_2a'_), 2.03 (t, *J* = 0.6 Hz, 4H, Me_3a'_, H_12''_), 1.88 (m, 3H, H_1''_, H_2'_, H_7''_), 1.69 (m, 1H, H_25_), 1.49 (m, 3H, H_7'_, H_11''_, H_15''_), 1.45 (m, 1H, H_8_), 1.22 (m, 14H, H_2''_, H_11'_, H_12'_, H_14_, H_15'_, H_16_, H_17_, H_20_, H_22_, H_23''_, H_28_), 1.02 (m, 4H, Me_19_, H_1'_), 0.94 (m, 4H, Me_21_, H_24_), 0.89 (s, 1H, H_9_), 0.87 (d, *J* = 0.9 Hz, 3H, Me_29_), 0.85 (m, 3H, Me_26_), 0.84 (m, 3H, Me_27_), 0.70 (m, 4H, Me_18_, H_23'_).

*(2R,3R,4R,5R,6R)-2-(((3S,8S,9S,10R,13R,14S,17R)-17-((2R,5R)-5-ethyl-6-methylheptan-2-yl)-10,13-dimethyl-2,3,4,7,8,9,10,11,12,13,14,15,16,17-tetradecahydro-1H-cyclopenta[a]phenanthren-3-yl)oxy)-6-(hydroxymethyl)tetrahydro-2H-pyran-3,4,5-triol* (***b*-1**)

*
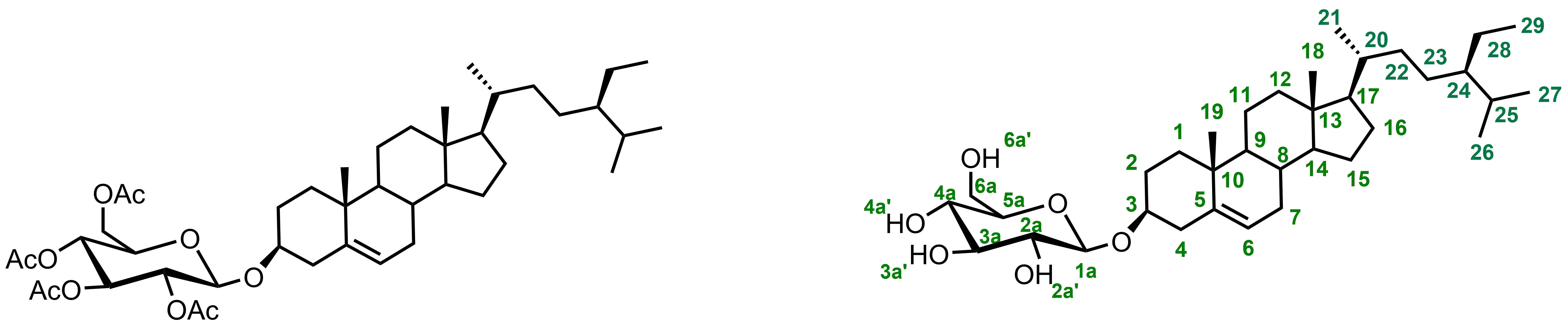
*

**β-1**

A fresh solution of MeONa prepared starting from Na (120 mg, 5.2 mmol) in 18 mL of MeOH was added to a solution of **β-2** (3.35 g, 4.4 mmol) in MeOH (18 mL) and DCM (72 mL). After 3 h at rt, Dowex 50w X 8 (H^+^) acidic resin (33 g) was added, and the reaction was stirred for an additional 2 h. The resin was then removed by filtration, the organic phase was concentrated to dryness and the solid obtained was washed with MeOH several times. The crude product isolated after the first filtration was 2.2 g. The solid was triturated for two days in AcOEt, to give after filtration a product as white solid (1.65 g 63%, purity 98.1%, m.p.: 280 °C).

^1^H NMR (400 MHz, DMSO) δ 5.32 (m, 1H, H_6_), 4.85 (m, 3H, H_2a'_, H_3a'_, H_4a'_), 4.40 (t, *J* = 5.8 Hz, 1H, H_5a'_), 4.22 (d, *J* = 7.8 Hz, 1H, H_1a_), 3.64 (dd, *J* = 5.9, 10.9 Hz, 1H, H_6a'_), 3.43 (m, 2H, H3, H_6a''_), 3.12 (m, 1H, H_3a_), 3.03 (dq, *J* = 4.9, 13.2 Hz, 2H, H_4a_, H_5a_), 2.89 (td, *J* = 4.4, 8.2 Hz, 1H, H_2a_), 2.37 (m, 1H, H_4'_), 2.12 (t, *J* = 12.4 Hz, 1H, H_4''_), 1.94 (m, 3H, H_2'_, H_7''_, H_12'_), 1.81 (m, 2H, H_16'_, H_16''_), 1.78 (d, *J* = 4.0 Hz, 1H, H_1‘),_ 1.62 (dt, *J* = 6.7, 13.0 Hz, 1H, H25), 1.52 (m, 2H, H2'', H15''), 1.47 (m, 1H, H11'), 1.39 (m, 3H, H7', H8, H_11''_), 1.35 (s, 1H, H_20_), 1.28 (m, 1H, H_28''_), 1.22 (t, *J* = 8.0 Hz, 1H, H_28'_), 1.15 (m, 3H, H_12''_, H_23_), 1.09 (m, 1H, H_17_), 1.01 (m, 3H, H_1''_, H_15'_), 0.96 (s, 3H, Me_19_), 0.90 (m, 8H, Me_21_, H_9_, H_14_, H_22_, H_24_), 0.81 (m, 9H, Me_29_, Me_26_, Me_27_), 0.65 (s, 3H, Me_18_).

^13^C{^1^H} NMR (C100 MHz, DMSO) δ 140.9 (C_5_), 121.7 (C_6_), 101.3 (C_1a_), 77.4 (C_3_), 77.2 (C_3a_), 77.2 (C_5a_), 73.9 (C_2a_), 70.6 (C_4a_), 61.6 (C_6a_), 56.7 (C_14_), 55.9 (C_17_), 50.1 (C_9_), 45.6 (C_24_), 42.3 (C_13_), 39.8 (C_12_), 38.8 (C_4_), 37.3 (C_1_), 36.7 (C_10_), 36.0 (C_20_), 33.8 (C_22_), 31.9 (C_8_), 31.8 (C_7_), 29.7 (C_2_), 29.2 (C_25_), 28.3 (C_16_), 25.9 (C_23_), 24.3 (C_15_), 23.1 (C_28_), 21.1 (C_11_), 20.2 (Me_27_), 19.6 (Me_19_), 19.4 (Me_26_), 19.1 (Me_21_), 12.3 (Me_29_), 12.1 (Me_18_).

ESI-MS (*m/z*): [M+Na^+^]. Calculated for C_35_H_60_NaO_6_^+^ 599.4282; Found 599.4247

^1^H NMR of **β-2** (CDCl_3_)


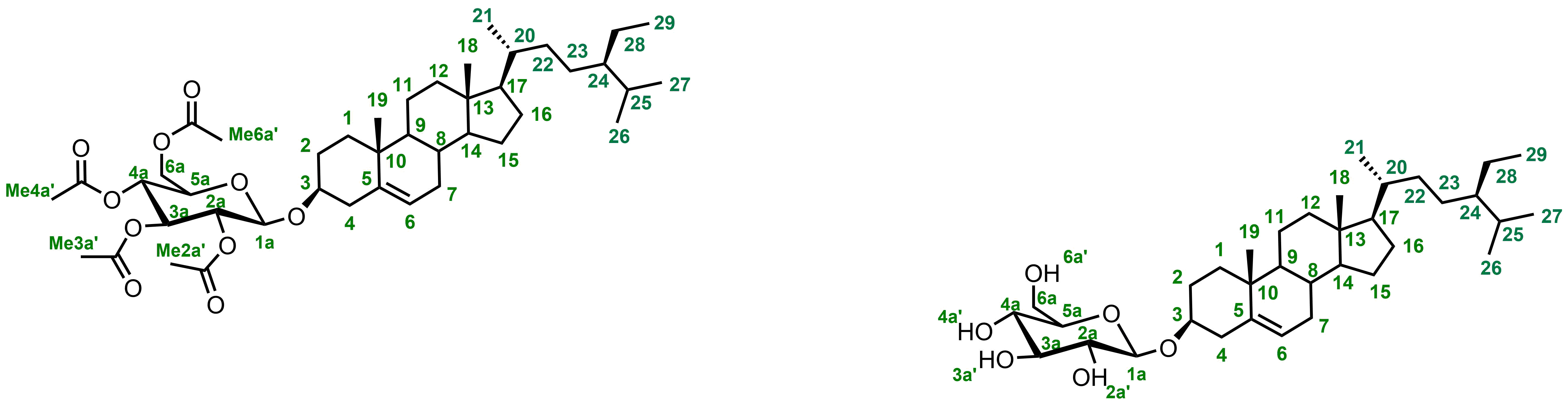

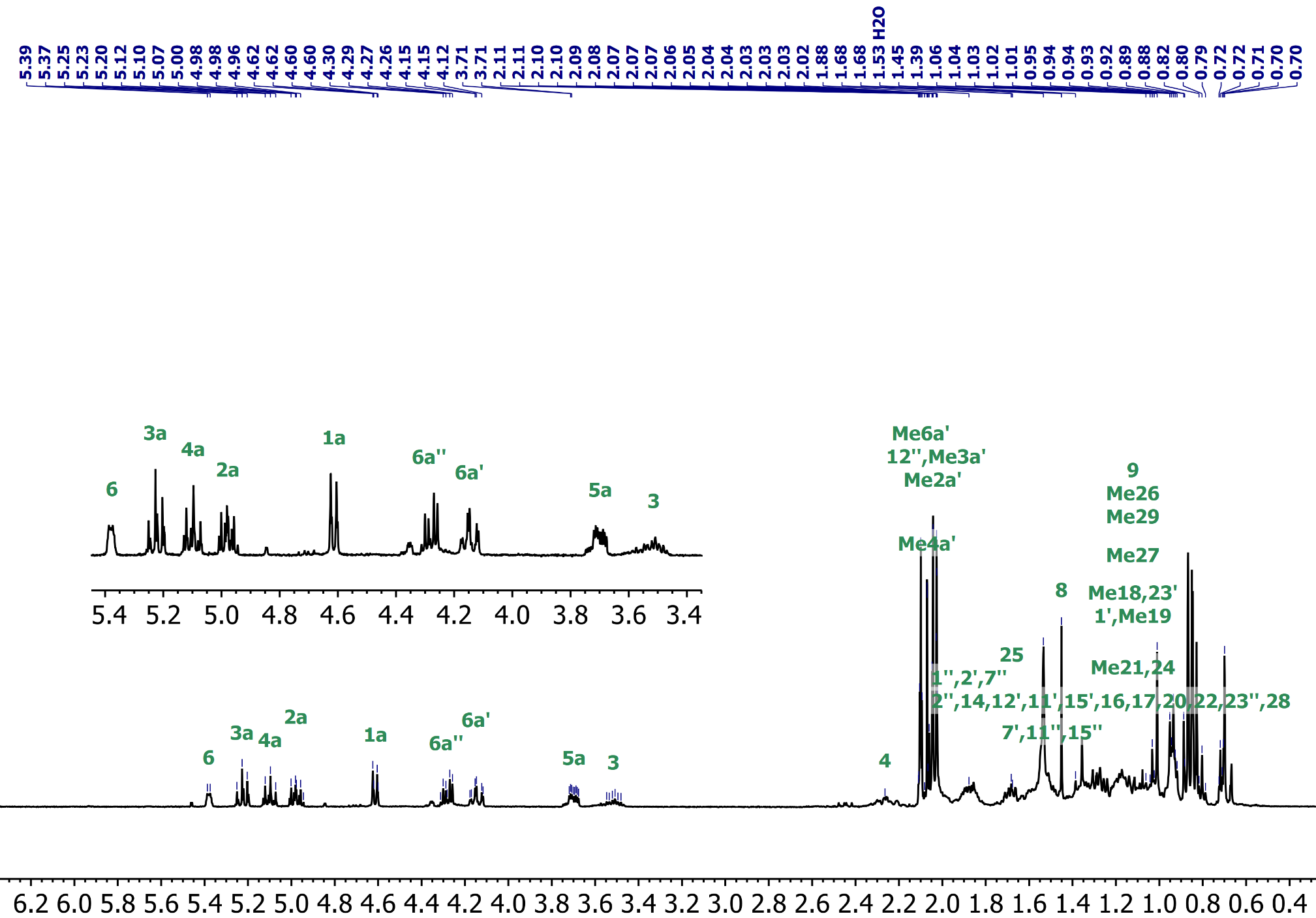


^1^H NMR of **β-1** (DMSO-_d6_)


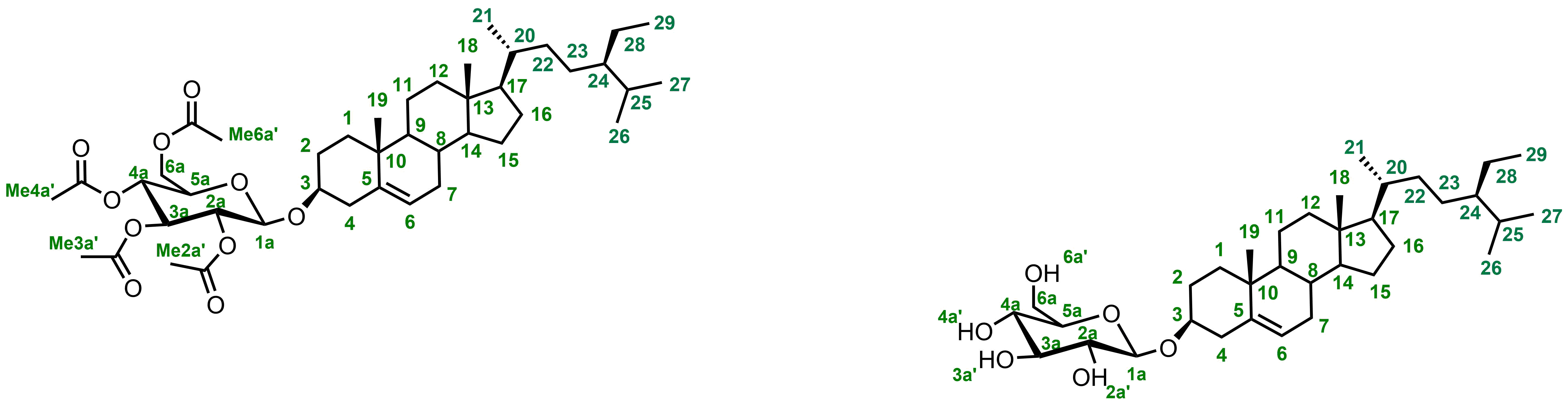

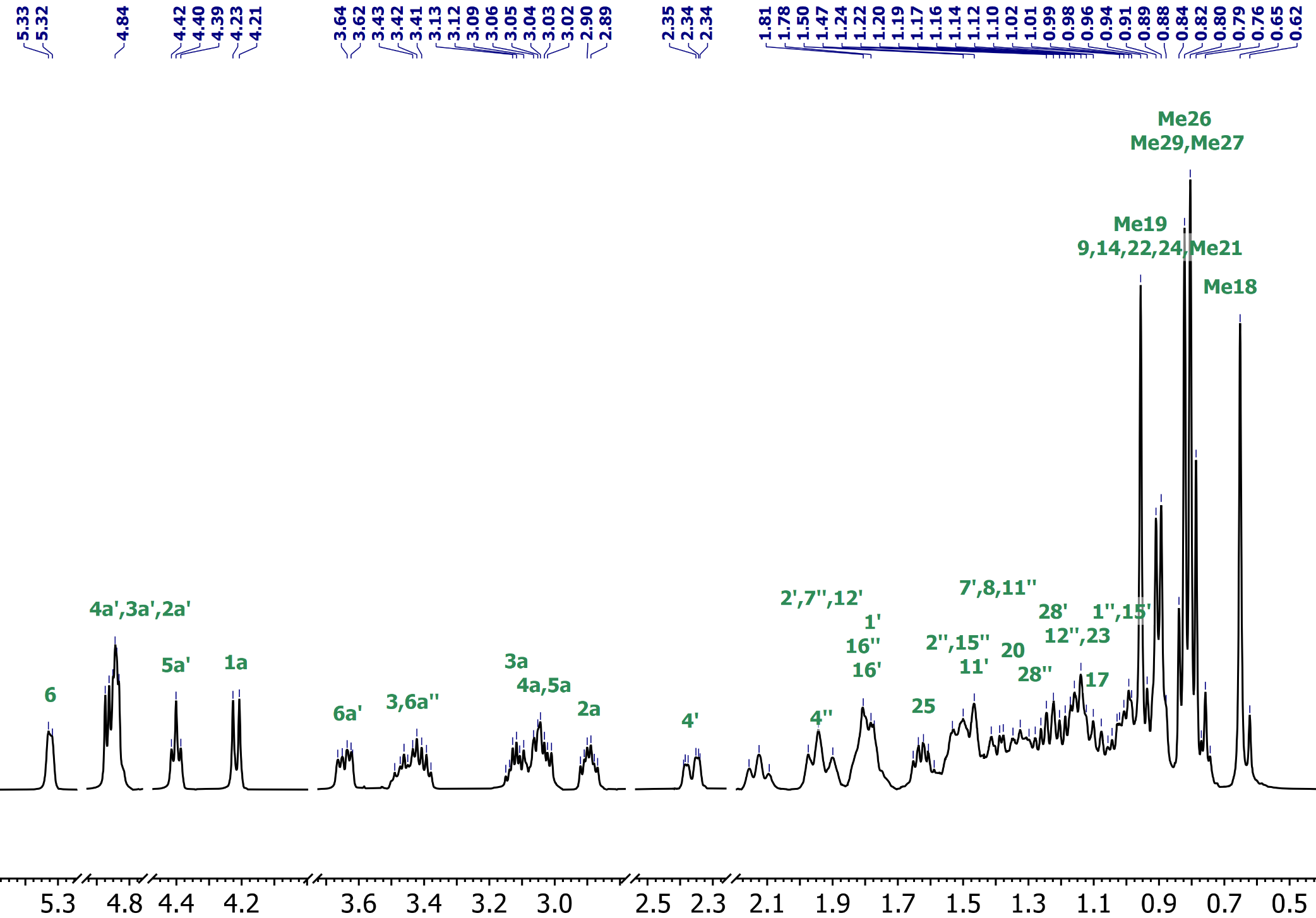


^13^C{1H})of **β-1** (DMSO-_d6_


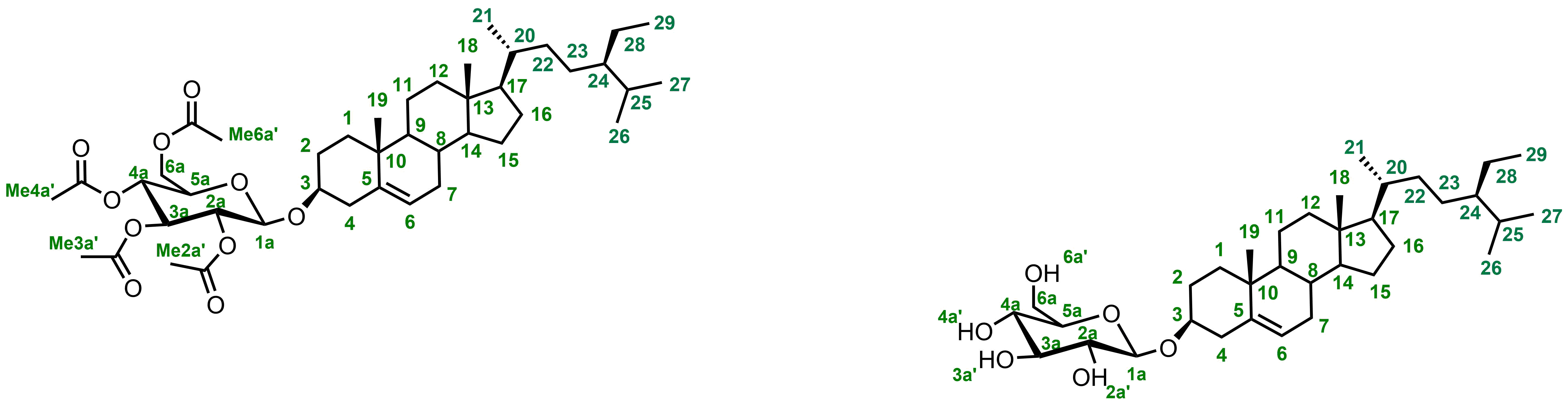

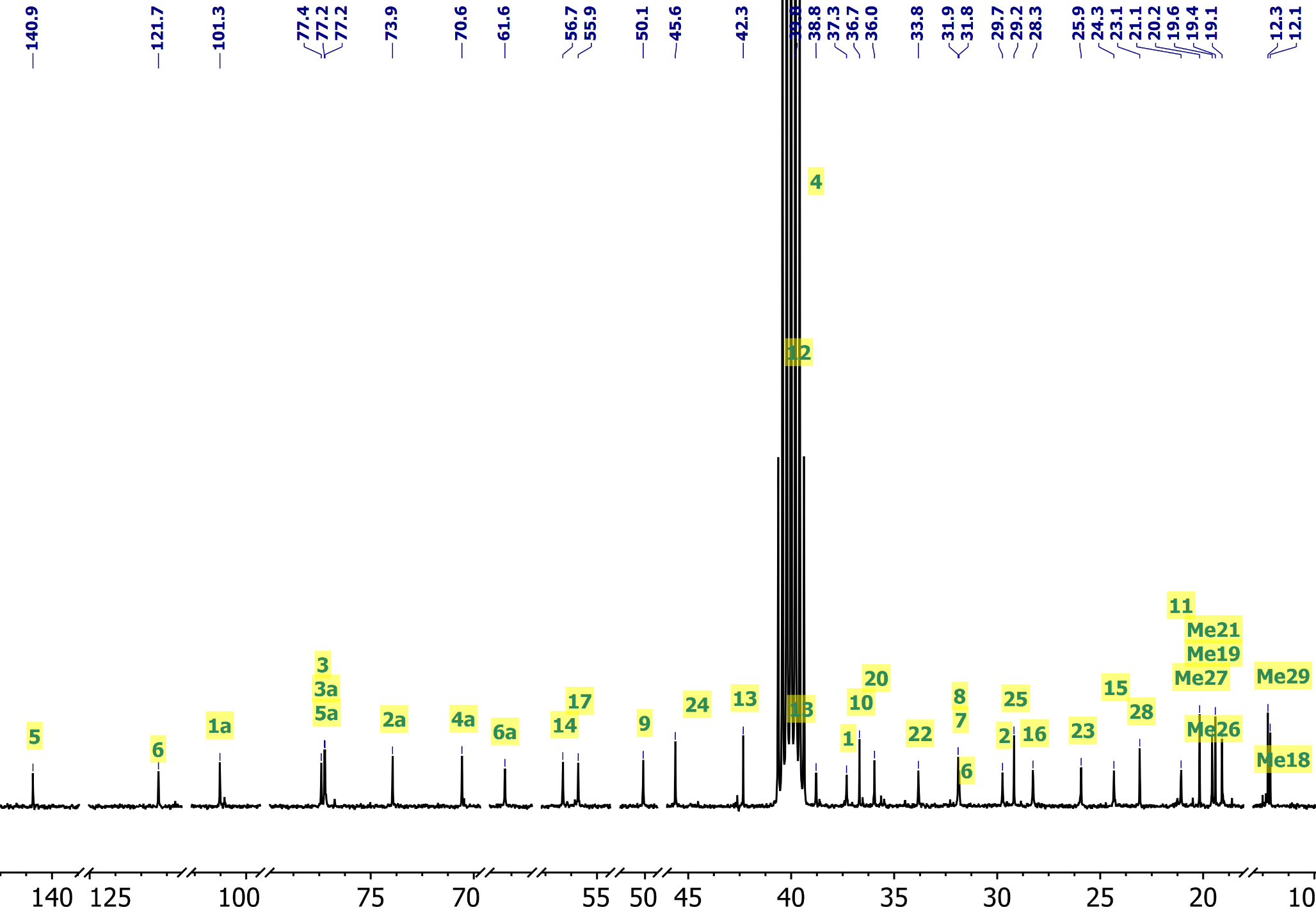


COSY of **β-1** (DMSO-_d6_)


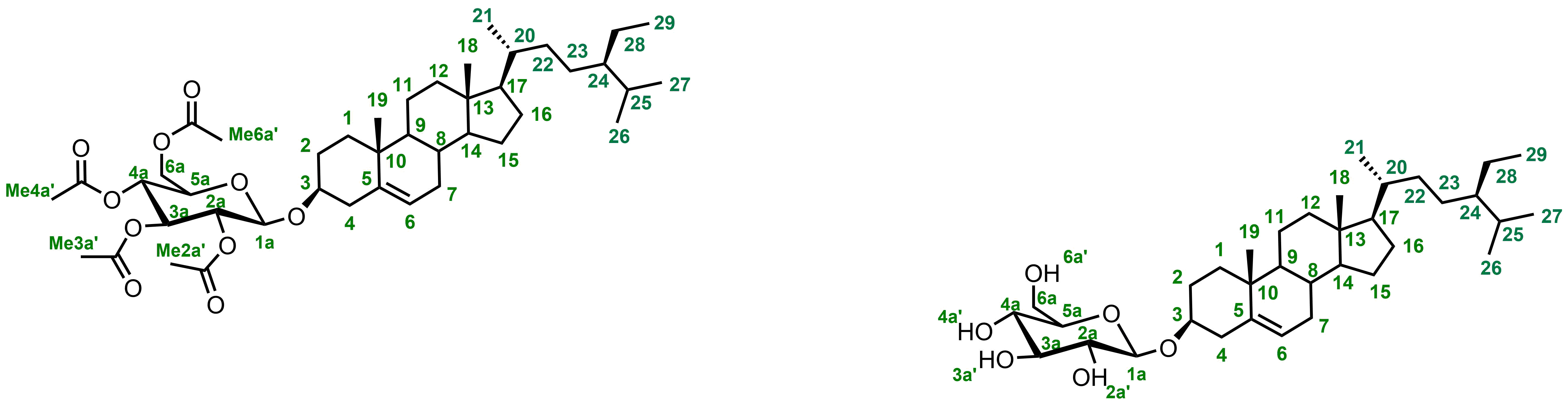

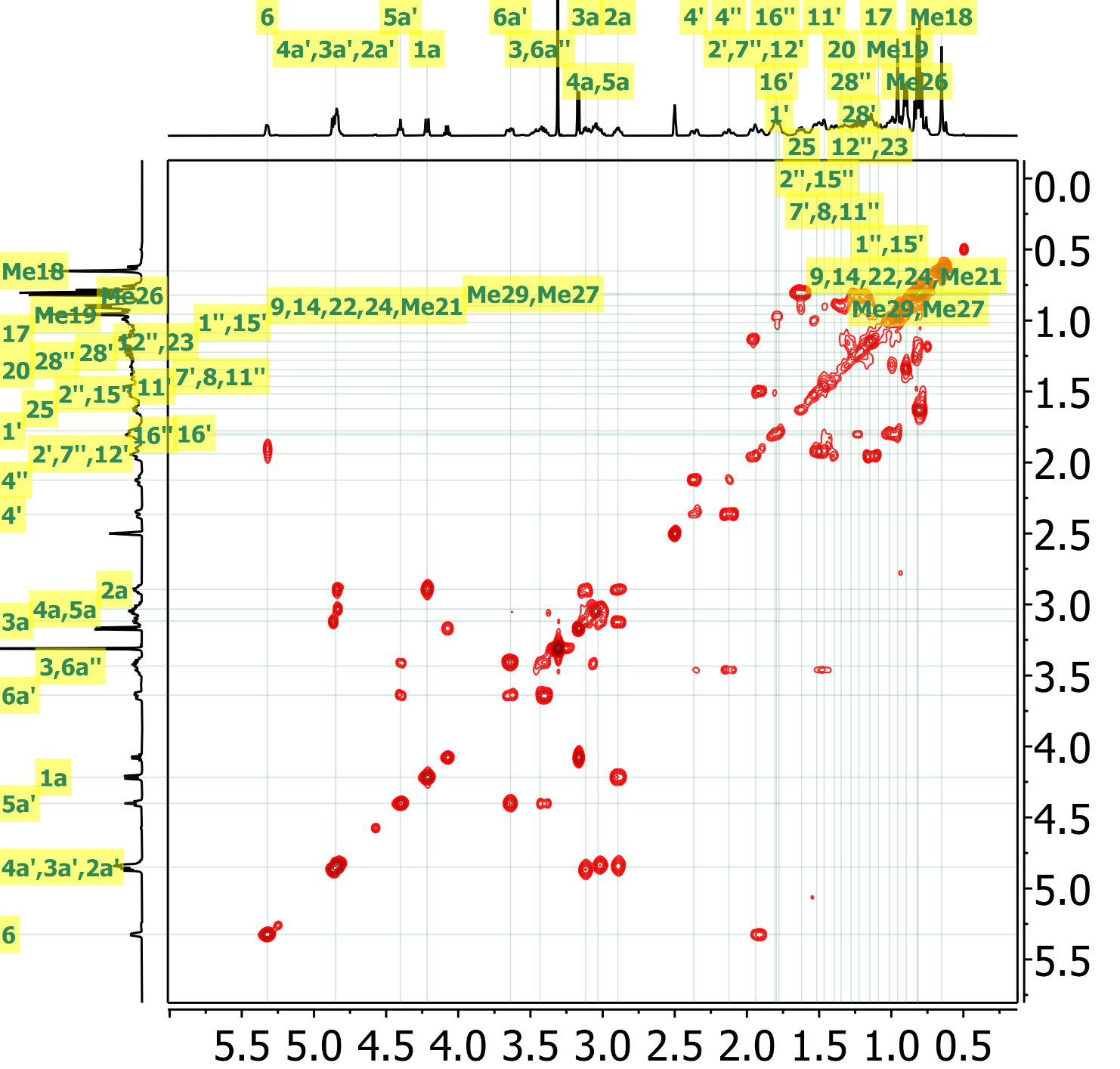


NOSY of **β-1** (DMSO-_d6_)


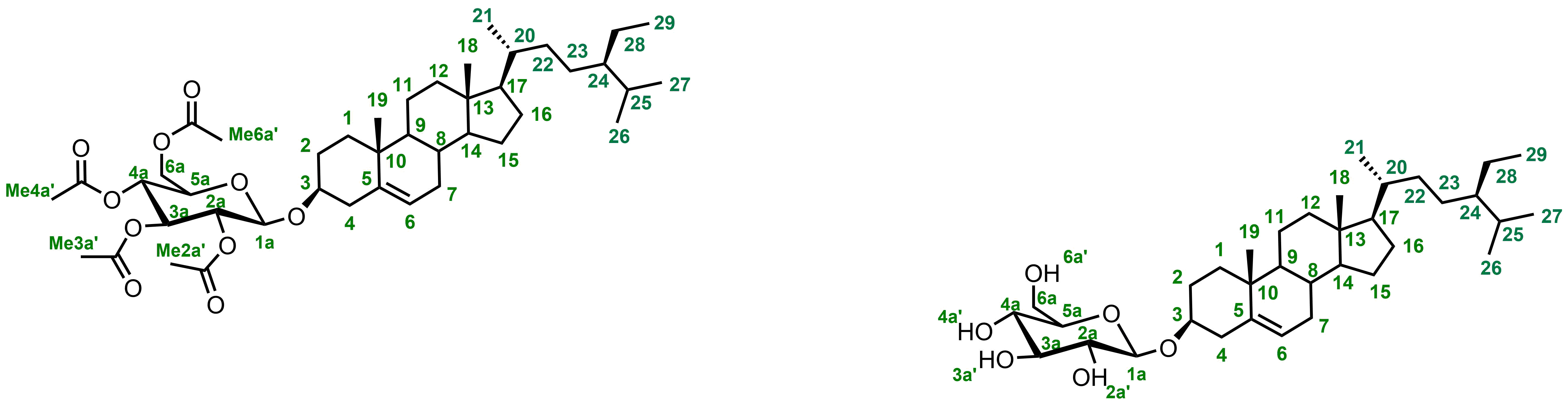

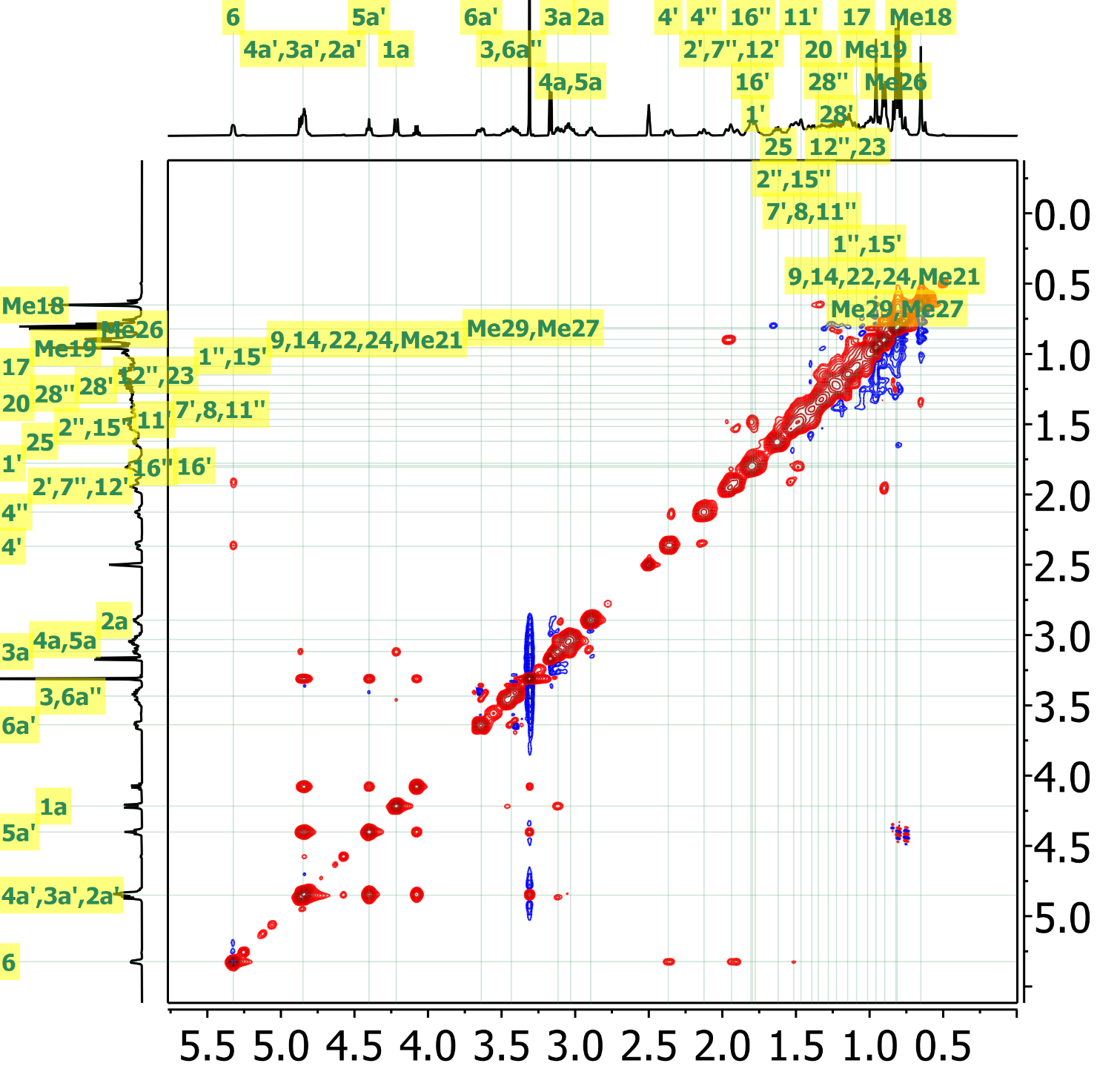


HSQC of **β-1** (DMSO-_d6_)


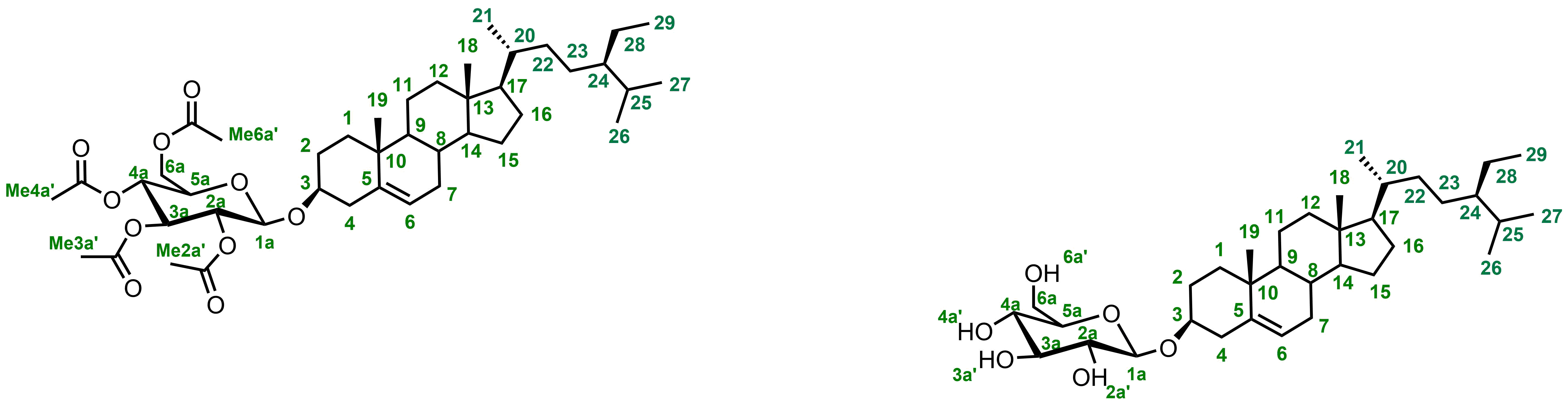

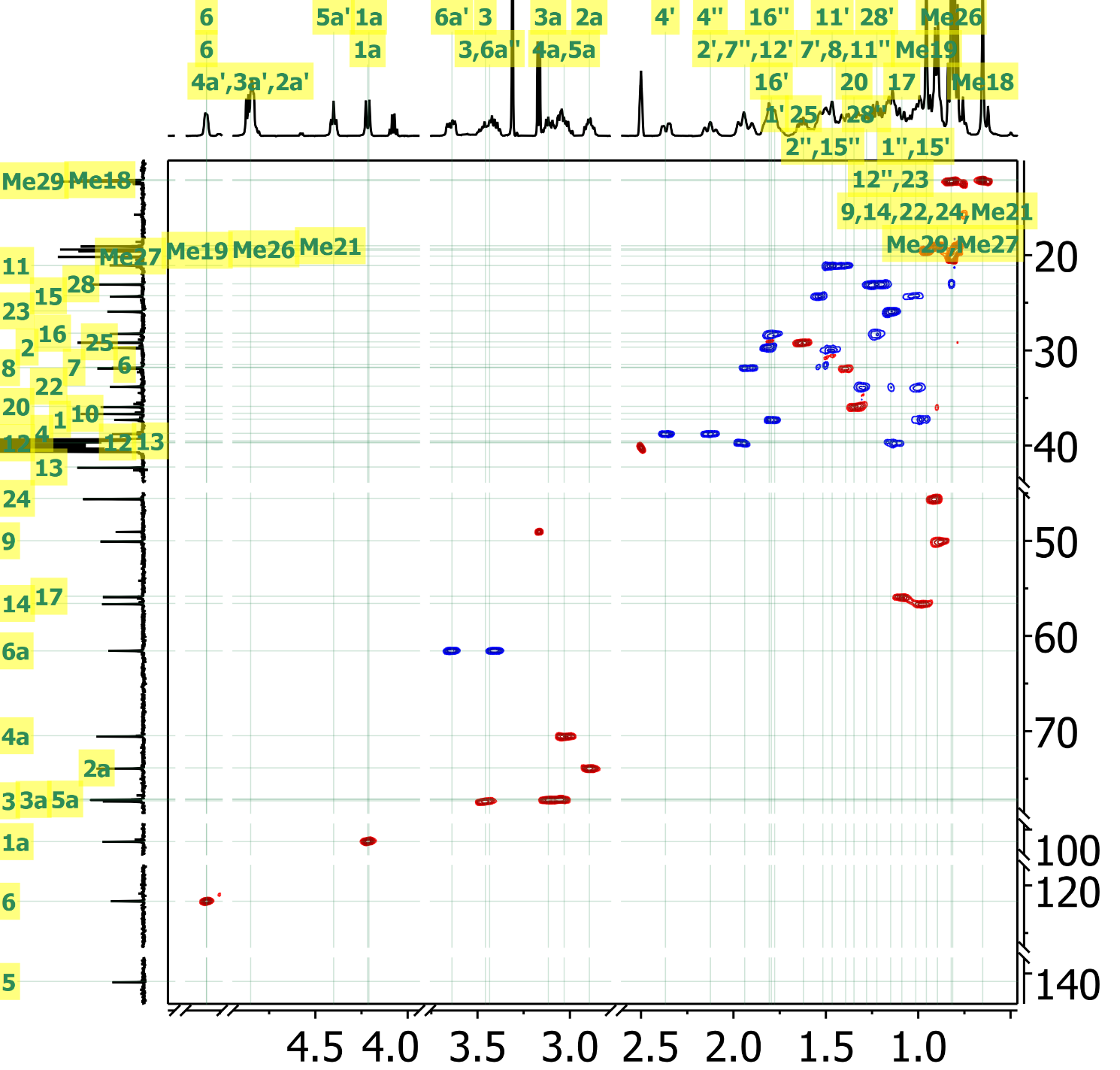


HMBC of **β-1** (DMSO-_d6_)


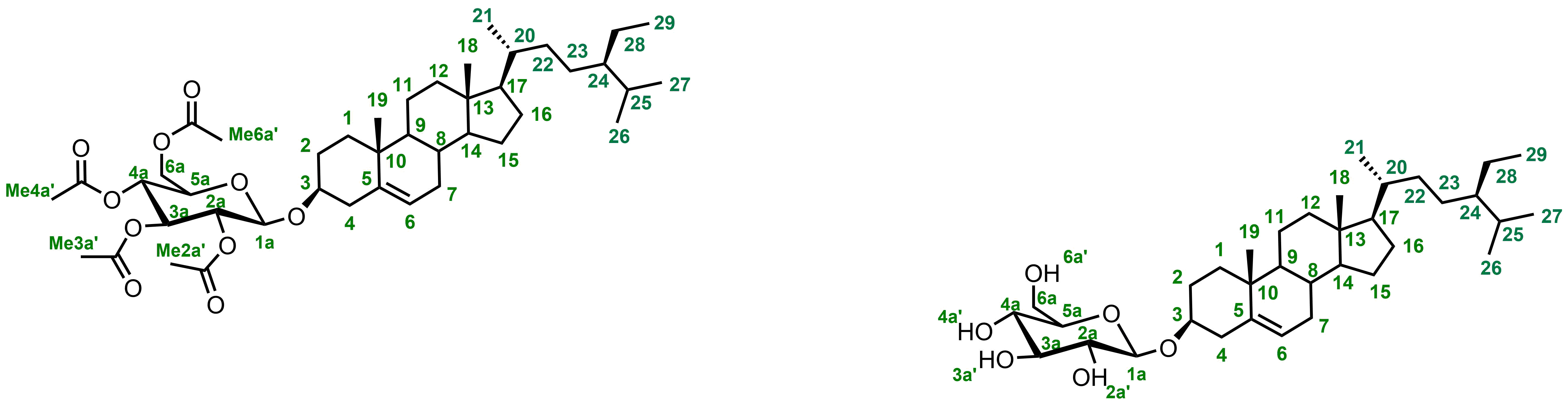

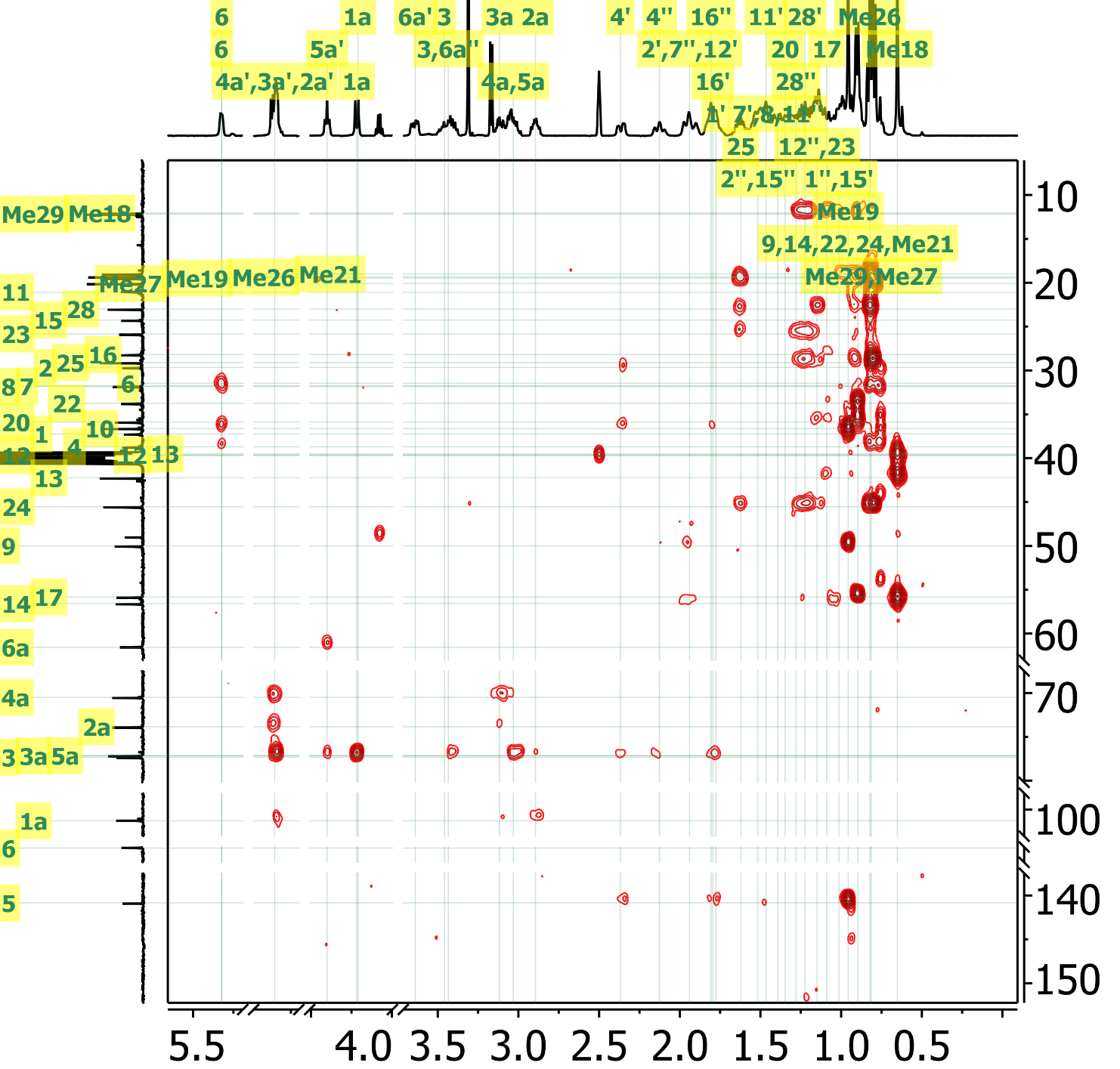


HRMass of **β-1**


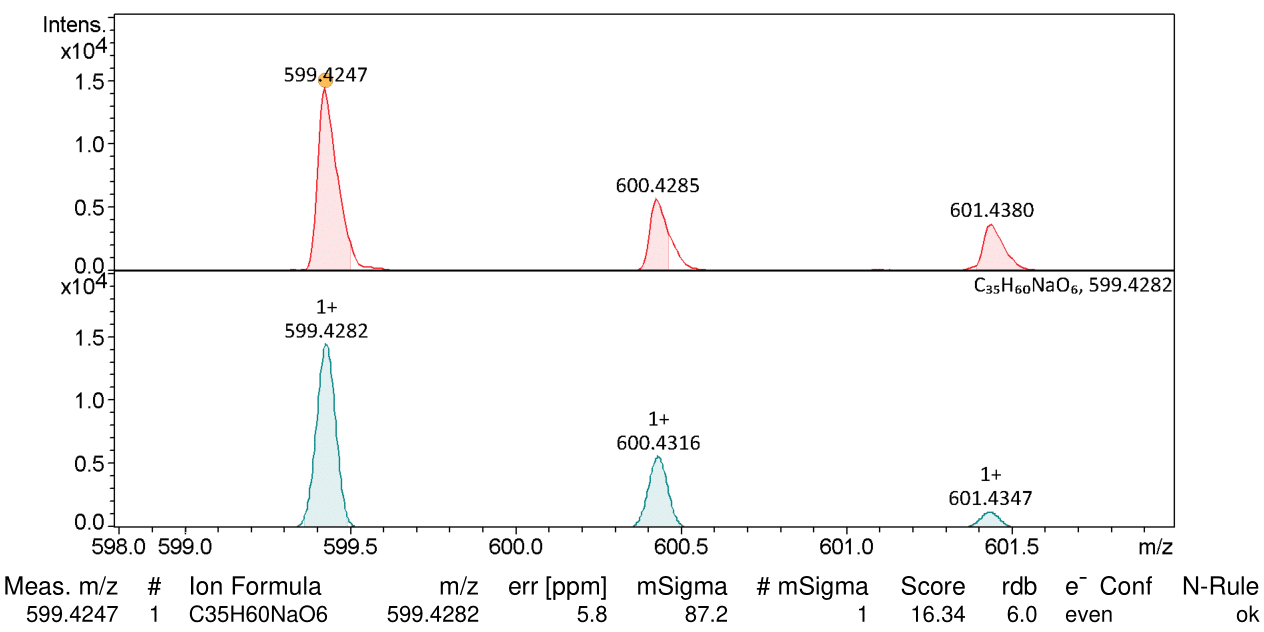


**Simulated Spectra**

**Real Spectra**


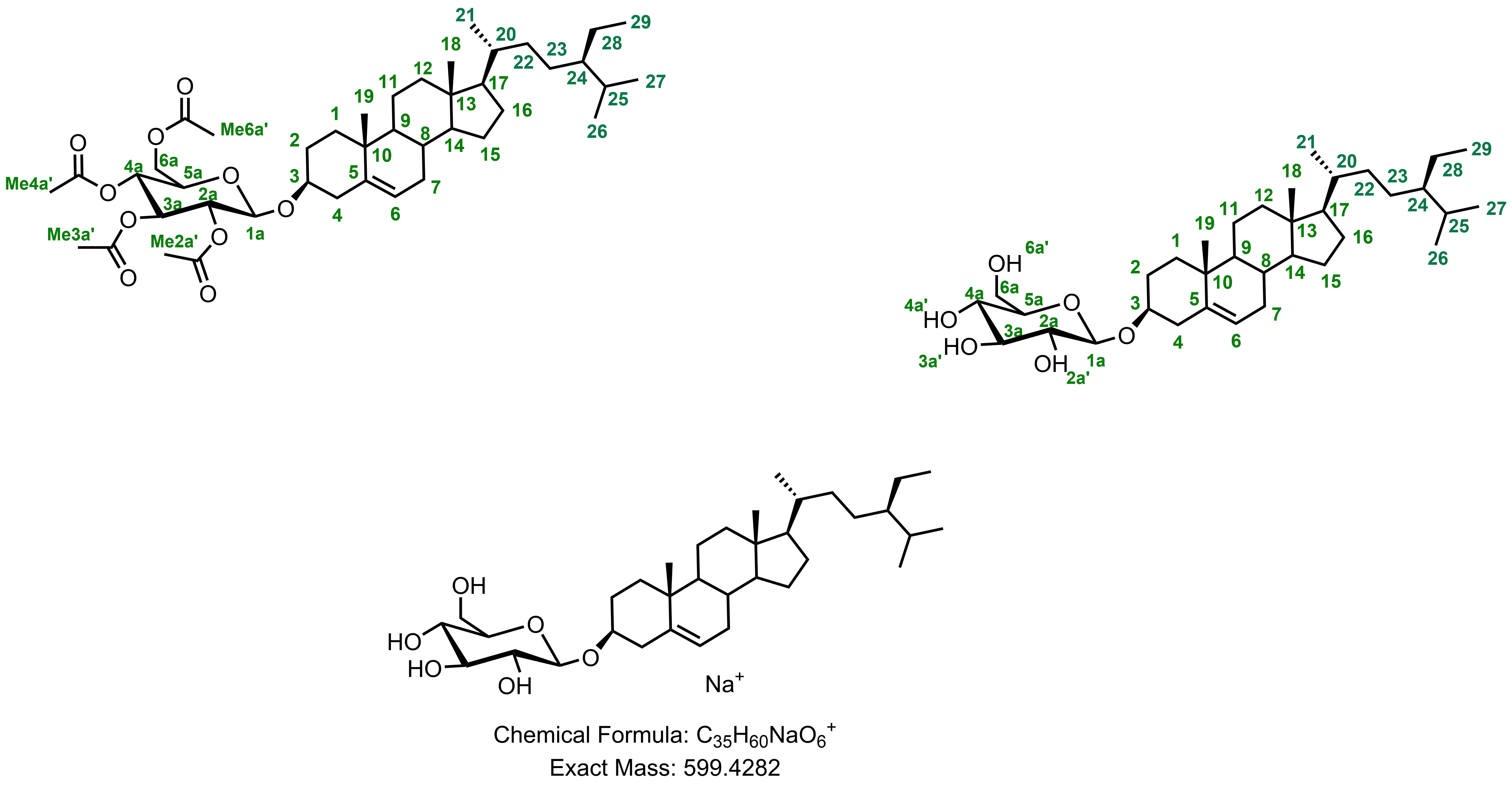


qNMR of **β-1**


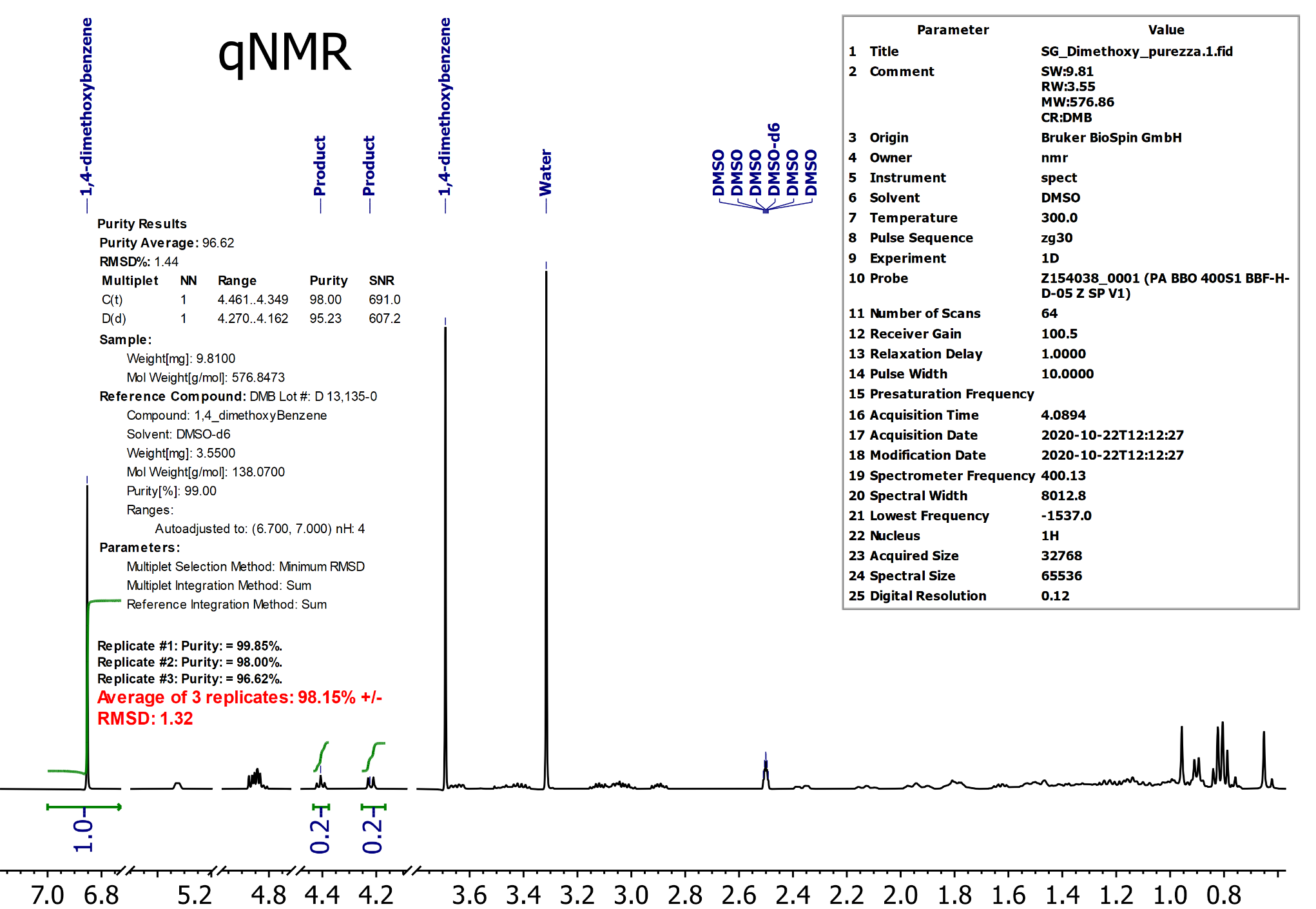


Bibliography

1. . Rubnov, S.; Kashman, Y.; Rabinowitz, R.; Schlesinger, M.; Mechoulam, R., *J. Nat. Prod.* **2001**, *64*, 993. [↑](#endnote-ref-1)
2. . Maslov, M. A.; Morozova, N. G.; Chizhik, E. I.; Rapoport, D. A.; Ryabchikova, E. I.; Zenkova, M. A.; Serebrennikova, G. A., *Carbohydr. Res.* **2010**, *345*, 2438 [↑](#endnote-ref-2)
3. . Mura Faizi, S.; Ali, M.; Saleem, R.; Irfanullah; Bibi, S., *Magn. Reson. Chem.* **2001**, *39*, 399 [↑](#endnote-ref-3)
4. . (a) Dominquez S*. Mnova qNMR training*; (b) Bernstein, M. *Purity by NMR: making it robust and automated.* Retrieved from https://mestrelab.com/learn_support/mnova/qnmr/ [↑](#endnote-ref-4)
5. . W. L. F. Armarego in *Purification of Laboratory Chemicals*, 18th edition, Elsevier, **2017**. [↑](#endnote-ref-5)
6. . W. C. Still, M. Kahn, A. Mitra, *J. Org. Chem.* **1978**, *43*, 2923-2925. [↑](#endnote-ref-6)
