# Supplementary material for: β-Sitosterol β-d-glucoside (BSSG) triggers intestinal inflammation in zebrafish and mouse models prior to neurodegeneration onset
Source: J Biomed Sci. 2026 May 4;33:45. doi: 10.1186/s12929-026-01249-8 (PMC13137512; doi:10.1186/s12929-026-01249-8)
Supplement: Supplementary file 2 — Additional file 2. Primers tables. Primers. [file 12929_2026_1249_MOESM2_ESM.docx]

**Table S1. Primers used for the genotyping of zebrafish mutant lines.**

| Gene | Forward sequence | Reverse sequence |
| --- | --- | --- |
| *gr* | ACCACTTCAAGCGGACAGAG | CCGGCTTCTGATCTTTCTGC |
| *cyp11c1* | TTCACCAAAGCTCCAGATCC | ACTCCTCCATGTGTTTGTGC |

**Table S2. Primers used for the amplification of bacterial DNA.**

| Primers PCR I | Primer sequence | Amplicon length |
| --- | --- | --- |
| Eub8F (forward) | AGAGTTTGATCMTGGCTCAG | 976 bp |
| 984yR (reverse) | GTAAGGTTCYTCGCGT |  |

| Primers PCR II | Primer sequence | Amplicon length |
| --- | --- | --- |
| 515F (forward) | GTGYCAGCMGCCGCGGTAA | 358 bp |
| 806R (reverse) | GGACTACNVGGGTWTCTAAT |  |

Primers used for the amplification of bacterial DNA extracted from zebrafish gut. Primers PCR II were used also for the amplification of hypervariable region V4 of bacterial DNA extracted from mouse fecal samples.

**Table S3. Zebrafish and mouse primers used in RT-qPCR analyses.**

| Zebrafish genes | Forward sequence | Reverse sequence |
| --- | --- | --- |
| *β-actin* | TGGGTATGGAATCTTGCGGT | GTGGGGCAATGATCTTGATC |
| *ef1α* | TTCGAGAAGGAAGCCGCTG | CAGCAACAATCAGCACAGCAC |
| *agr2* | GCACAGACATACGAGGAAGC | GGAGACAAGTGCTTATCTGTG |
| *atg5* | CAGAGGATGTGGAGGAAATGTG | GAGTTTGAAGCATGGAGGTCG |
| *casp8* | GGGCAAAGCTGGGAAGATC | CTCCGTGTGAGAGAATACAGC |
| *fkbp5* | GTGTTCGTCCACTACACC | TCTCCTCACGATCCCACC |
| *foxo3b* | CTTCAAGGAGGAATGCATGG | TTATGCAGGGACAGGTTATGC |
| *il-4* | GCAGGAATGGCTTTGAAGGG | TCCTTCATTGTGCATTCCCC |
| *il-13* | TTTCTGTCAGGCTGAGGAGG | CGTCTTGGTGGTTGTAAGTGT |
| *lc3b* | GGTGGAGGATGTACGGCT | CTCATGTTAACGTGGTCAGG |
| *mmp9* | CATTAAAGATGCCCTGATGTATCCC | AGTGGTGGTCCGTGGTTGAG |
| *mmp13* | ATGGTGCAAGGCTATCCCAAGAGT | GCCTGTTGTTGGAGCCAAACTCAA |
| *nupr1* | AAAACATCGAGCCGACCACC | CTCCTTCTTCGTCCTCCC |
| *pept1* | GATTGCTTTGGGAACAGGAGG | GATGGGTGTGATGAGAGTGG |
| *p62* | TCTGACGATGAGCTAATGATGG | CTAAAGTGAGGTGTAGTGAACG |
| *stat3* | CTACTCCGGTTGCCAGATCA | GGCTTTGGACTCAGGATTGC |
| Mouse genes |  |  |
| *Tbp* | GATCAAACCCAGAATTGTTCTCC | TATGTGGTCTTCCTGAATCCCT |
| *Il-1β* | TGGCTGTGGAGAAGCTGTG | CATGAGTCACAGAGGATGGG |
| *Ifn-γ* | GAAAGACAATCAGGCCATCAGC | ATTGAATGCTTGGCGCTGGAC |
| *Ikb-α* | GAAGCAGCAGCTCACGGAG | AAGTTGAGGAAGGCCAGGTC |
| *Nlrp3* | GATCAACAGGCGAGACCTC | CACTCTTCTTCAAGGCTGTC |
| *Nod1* | TCAGGCTGTGGCTCTCAGA | GTTGGTGTCGCAGCTGTTG |
| *Plp-1* | GTCTCTGCGCTGATGCCAG | CGCAGCACCCACAAACGC |
| *Reg3-γ* | GCTCCATGACCCGACACTG | GTACCACAGTGATTGCCTGAG |
| *Tlr2* | CAGACGCTGGAGGTGTTGG | ACAGGGAACAACGAAGCATCTG |
| *Tlr4* | TTCTTCTCCTGCCTGACACC | TTTGCTGAGTTTCTGATCCATGC |
| *Tlr6* | GGAGACAGCACTGAAGTCAC | GGTGTCTGAGATAGAGAGCATC |
