# Supplementary material for: β-Sitosterol β-d-glucoside (BSSG) triggers intestinal inflammation in zebrafish and mouse models prior to neurodegeneration onset
Source: J Biomed Sci. 2026 May 4;33:45. doi: 10.1186/s12929-026-01249-8 (PMC13137512; doi:10.1186/s12929-026-01249-8)
Supplement: Supplementary file 3 — Additional file 3. Figure S1. A) Fish embryo acute toxicologyTest on zebrafish larvae treated with increasing concentrations of BSSG and of the vehicle DMSO. B) Measure of larval morphological traits: standard length, eye area and area of the swimming bladder. N =3 biological replicates, each consisting of at least 10 larvae. Bar graphs show the mean ± SEM. Statistical analysis was performed using unpaired Student’s t-test. ns, not significant. C) Mass spectrometry analysis of lipid extracts from pooled heads and trunks of 5 dpf treated and control larvae. Numbers above the bars indicate the achieved internal concentration of BSSG. Figure S2. A) Magnification of the zebrafish mid-intestine region stained in vivo with neutral red and quantification of its length in larvae treated with 10 µM β-sitosterolcompared to controls. N = 4 biological replicates, each consisting of at least 10 larvae. Scale bar: 200 µm. B) RT-qPCR analysis of autophagy-related genes in pooled 5-dpf CTR and BSSG-treated larvae. N ≥3 biological replicates. Data are expressed as mean ± SEM. Statistical analysis was performed using unpaired Student’s t-test: *P<0.05; ***P<0.001. C) Immunofluorescence staining of HuC/D+ enteric neurons and Sox10+ neuronal progenitors in 5-dpf zebrafish larval intestine. Bar graphs show the mean ± SEM. Statistical analysis was performed using unpaired Student’s t-test. ns, not significant. Scale bar: 200 µm. D-E) Ex vivo concentration-response curves to carbachol stimulationand electric field stimulationin isolated ileal preparations of WTand mutant gr-/- adult zebrafishwith or without BSSG in vivo treatment. N ≥4 animals/condition. Statistical significance was calculated with a two-way ANOVA followed by a Bonferroni post hoc test for multiple comparisons. *P<0.05; ***P<0.001. Figure S3. A) Bar charts with RNAseq analysis from RNA samples of pooled 30 dpf chronically treated whole zebrafish larvae. GO Molecular function enrichment for the up- and downregulated genes. [file 12929_2026_1249_MOESM3_ESM.docx]

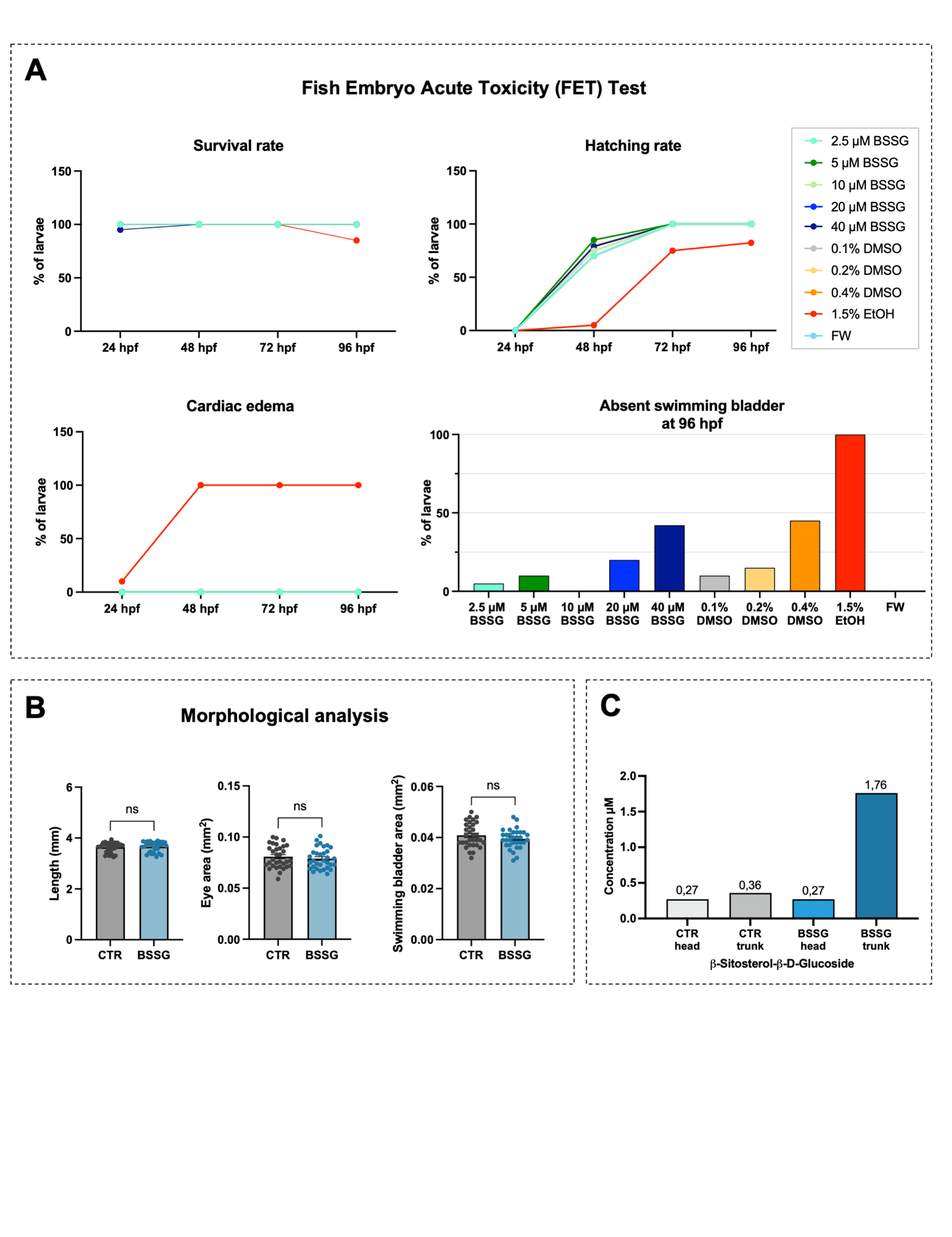


**Figure S1. A**) Fish embryo acute toxicology (FET) Test on zebrafish larvae treated with increasing concentrations of BSSG and of the vehicle DMSO. **B**) Measure of larval morphological traits: standard length, eye area and area of the swimming bladder. n=3 biological replicates, each consisting of at least 10 larvae. Bar graphs show the mean ± SEM. Statistical analysis was performed using unpaired Student’s t-test. *ns*, not significant. **C**) Mass spectrometry analysis of lipid extracts from pooled heads and trunks of 5 dpf treated and control larvae. Numbers above the bars indicate the achieved internal concentration of BSSG.


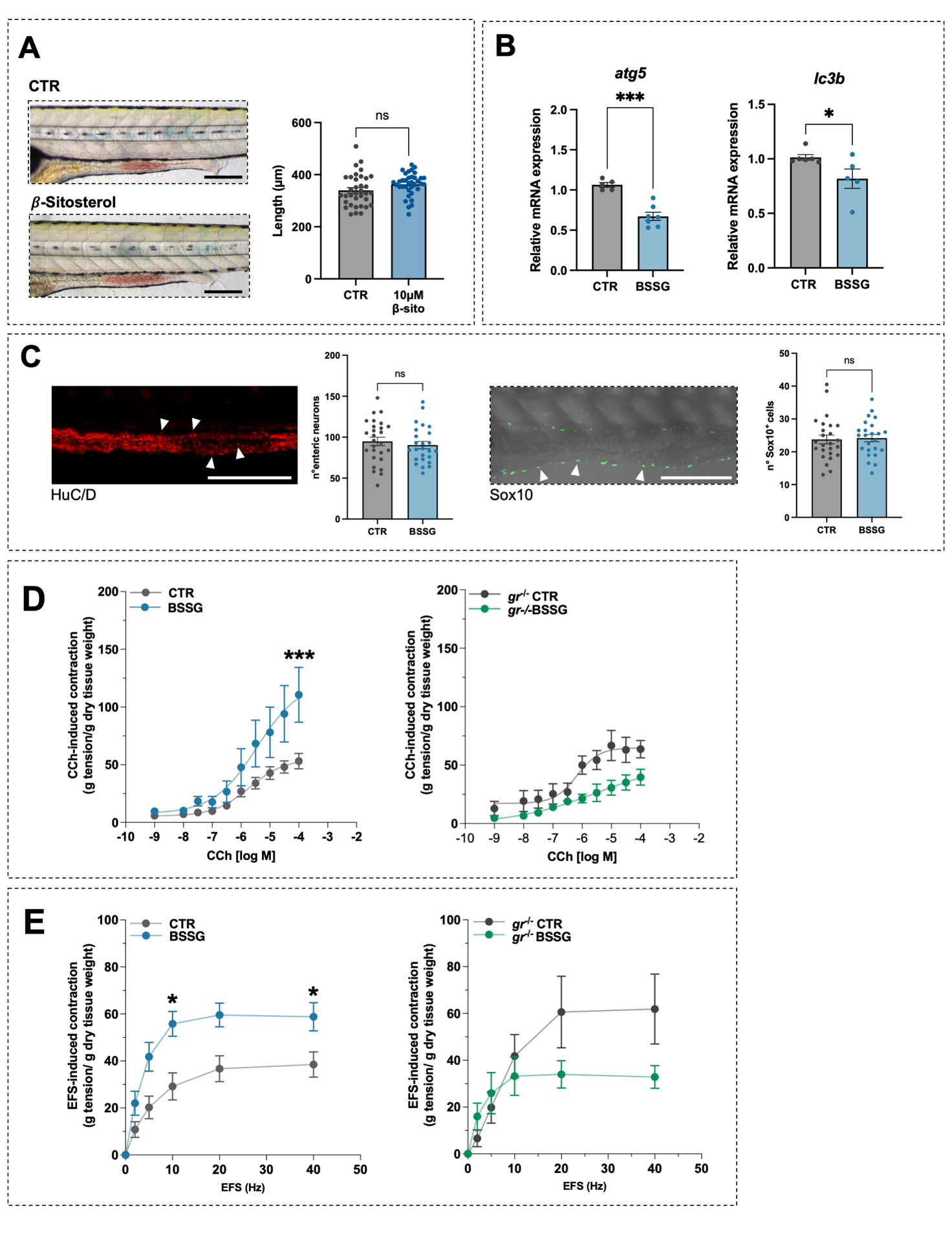


**Figure S2. A**) Magnification of the zebrafish mid-intestine region stained in vivo with neutral red and quantification of its length in larvae treated with 10 µM β-sitosterol (β-Sito) compared to controls. n= 4 biological replicates, each consisting of at least 10 larvae. Scale bar: 200 µm. **B**) RT-qPCR analysis of autophagy-related genes in pooled 5-dpf CTR and BSSG-treated larvae. n≥3 biological replicates. Data are expressed as mean ± SEM. Statistical analysis was performed using unpaired Student’s t-test: **P<0.05*; ****P<0.001*. **C**) Immunofluorescence staining of HuC/D+ enteric neurons and Sox10+ neuronal progenitors in 5-dpf zebrafish larval intestine (arrowheads). Bar graphs show the mean ± SEM. Statistical analysis was performed using unpaired Student’s t-test. ns, not significant. Scale bar: 200 µm. **D-E**) *Ex vivo* concentration-response curves to carbachol stimulation (0.001–100 μM) (**D**) and electric field stimulation (EFS; 0-40 Hz, 80V) (**E**) in isolated ileal preparations of WT (left panels) and mutant gr^-/-^ adult zebrafish (right panels) with or without BSSG *in vivo* treatment. N≥4 animals/condition. Statistical significance was calculated with a two-way ANOVA followed by a Bonferroni post hoc test for multiple comparisons. **P*<*0.05*; ***P<0.001.


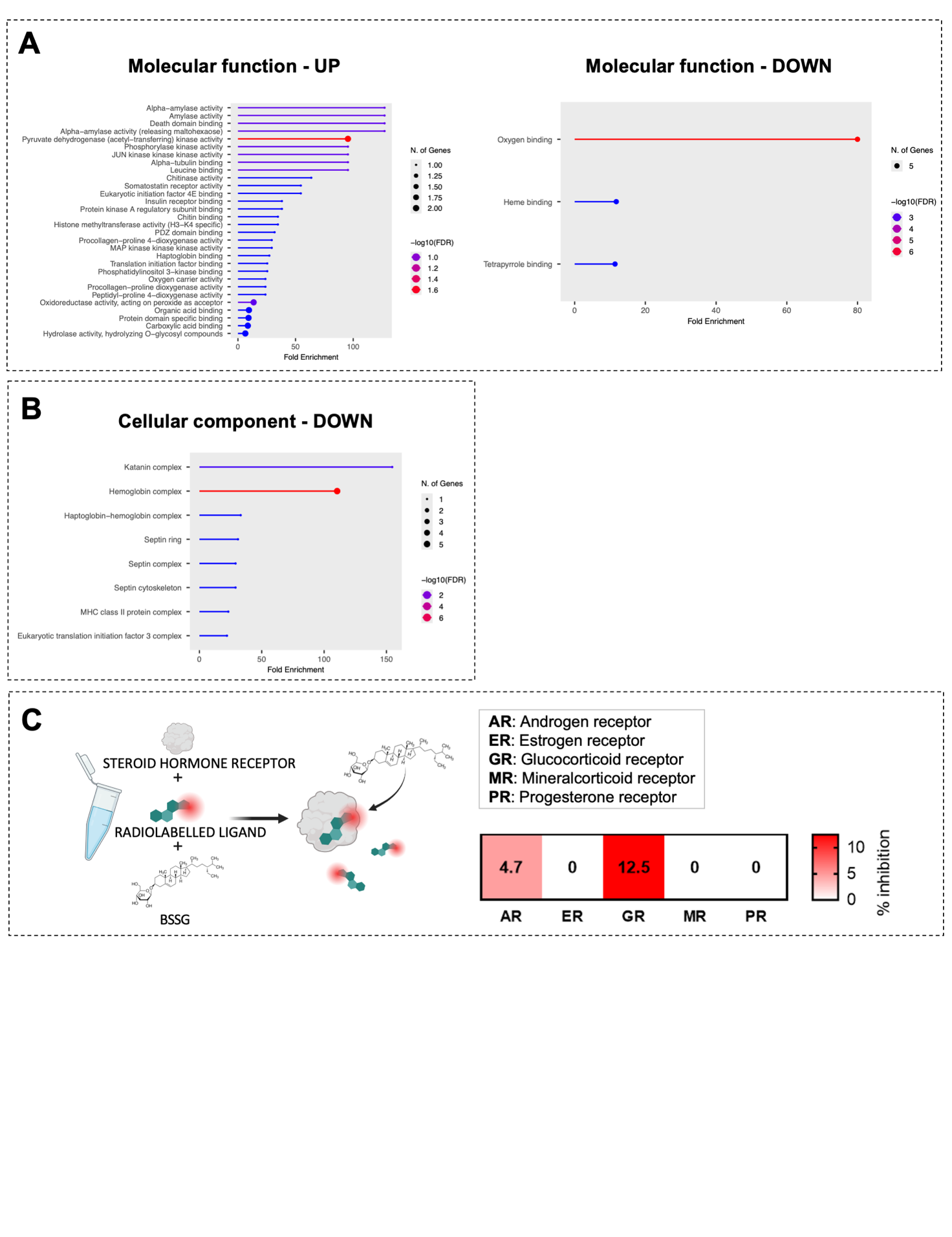


**Figure S3. A**) Bar charts with RNAseq analysis from RNA samples of pooled 30 dpf chronically treated whole zebrafish larvae. GO Molecular function enrichment for the up- and downregulated genes. **B**) GO Cellular component enrichment for downregulated genes. **C**) Schematic representation of the radioligand binding assay performed for each steroid hormone receptor with its specific radiolabelled ligand. The heatmap indicates the percentage of inhibition of GR binding to its radiolabelled specific ligand following the interaction with BSSG.


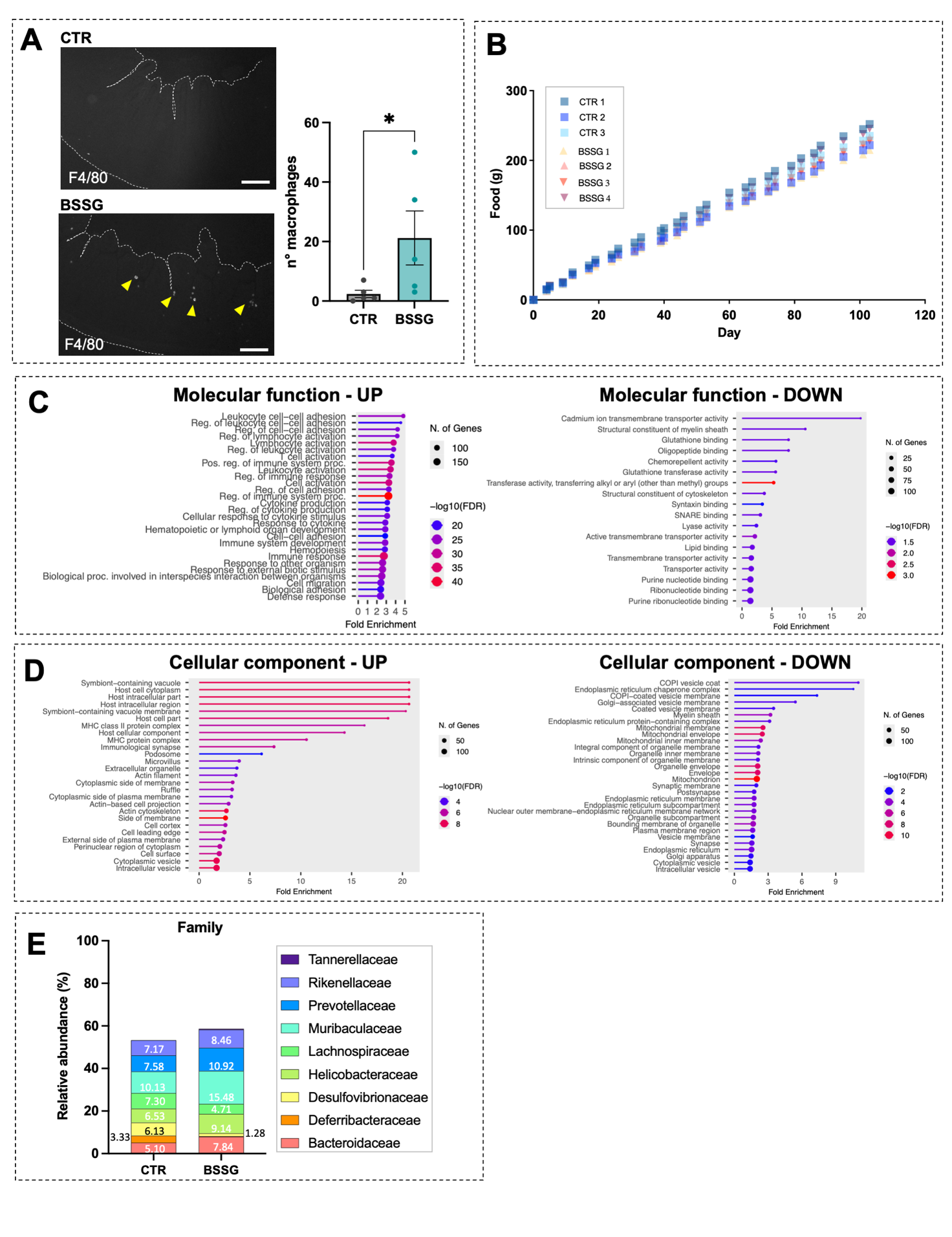


**Figure S4. A**) Immunofluorescence staining with the macrophage marker F4/80 on mouse small intestine histological sections. Arrowheads indicate macrophages in the *lamina propria*. Dotted lines define the outlines of the samples. Bar graph shows the mean ± SEM. Statistical analysis was performed on 5 sections obtained from 3 animals/condition using unpaired Student’s t-test. **P<*0.05. Scale bar: 200 µm. **B**) Representative plot of the cumulative amount of food (grams) consumed by BBSG-treated and control mice during the whole experimentation period. Food leftovers were weighed weekly to keep track of the amount of BSSG ingested by the animals through the feed. **C**)Bar charts with RNAseq analysis of RNA samples extracted from mouse small intestine. GO Molecular function enrichment of up- and downregulated genes. **D**) GO Cellular component enrichment of up- and downregulated genes. **E**) Analysis of mice fecal microbiota. The bar graph shows the percentage of relative abundance of bacterial families in N=2 animals/condition.


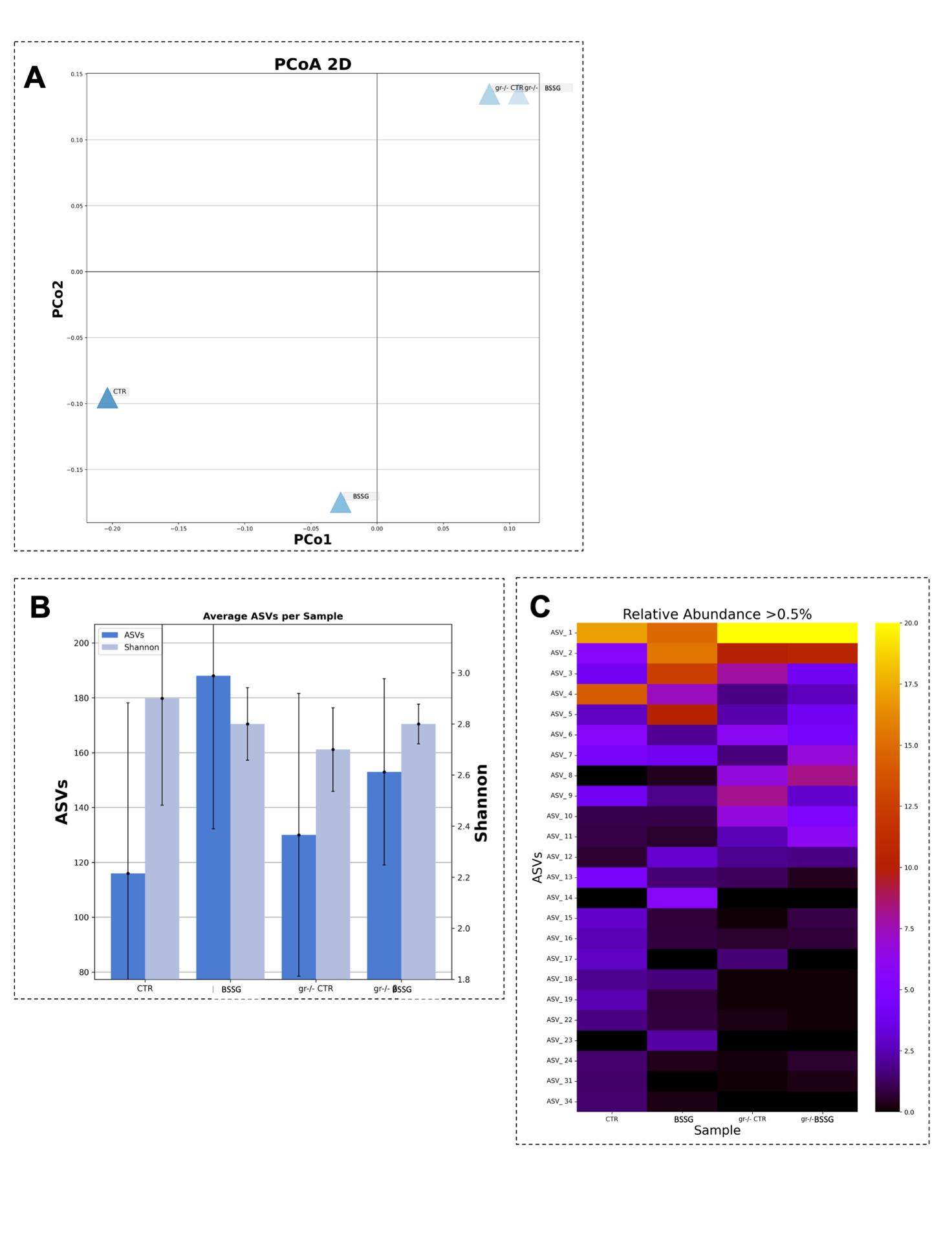


**Figure S5. A**) Principal Component analysis (PCOA_2D) of adult zebrafish gut microbiota analyzed in four different conditions: WT control (CTR), WT BSSG, *gr^-/-^* CTR and, *gr^-/-^* BSSG. The first (PCo1) and the second principal components (PCo2) are shown on the horizontal and vertical axes, respectively. **B**) Average ASVs in the four conditions. The number of ASVs and the Shannon index are plotted. **C**) Heatmap plotting the relative abundance of the ASVs that reach at least the 0.5%.
